# The microglial P2Y_6_ receptor mediates neuronal loss and memory deficits in neurodegeneration

**DOI:** 10.1016/j.celrep.2021.110148

**Published:** 2021-12-28

**Authors:** Mar Puigdellívol, Stefan Milde, Anna Vilalta, Tom O.J. Cockram, David H. Allendorf, Jeffrey Y. Lee, Jacob M. Dundee, Katryna Pampuščenko, Vilmante Borutaite, Hugh N. Nuthall, Jack H. Brelstaff, Maria Grazia Spillantini, Guy C. Brown

**Affiliations:** 1 Department of Biochemistry, University of Cambridge, Cambridge CB2 1QW, UK; 2 Department of Biomedicine, School of Medicine, Institute of Neuroscience, University of Barcelona, 08036 Barcelona, Spain; 3 Neuroscience Institute, Lithuanian University of Health Sciences, 50009 Kaunas, Lithuania; 4 Neuroscience, Eli Lilly Research & Development, Windlesham, Surrey GU20 6PH, UK; 5 Clinical Neurosciences, University of Cambridge, Cambridge CB2 0QQ, UK

**Keywords:** microglia, phagocytosis, neurodegeneration, P2Y_6_ receptor, memory deficits, Alzheimer’s disease, cell death

## Abstract

Microglia are implicated in neurodegeneration, potentially by phagocytosing neurons, but it is unclear how to block the detrimental effects of microglia while preserving their beneficial roles. The microglial P2Y_6_ receptor (P2Y_6_R) – activated by extracellular UDP released by stressed neurons – is required for microglial phagocytosis of neurons. We show here that injection of amyloid beta (Aβ) into mouse brain induces microglial phagocytosis of neurons, followed by neuronal and memory loss, and this is all prevented by knockout of P2Y_6_R. In a chronic tau model of neurodegeneration (P301S TAU mice), P2Y_6_R knockout prevented TAU-induced neuronal and memory loss. *In vitro*, P2Y_6_R knockout blocked microglial phagocytosis of live but not dead targets and reduced tau-, Aβ-, and UDP-induced neuronal loss in glial-neuronal cultures. Thus, the P2Y_6_ receptor appears to mediate Aβ- and tau-induced neuronal and memory loss via microglial phagocytosis of neurons, suggesting that blocking this receptor may be beneficial in the treatment of neurodegenerative diseases.

## Highlights

• P2Y_6_R knockout prevents microglial phagocytosis of stressed-but-viable cells
• P2Y_6_R knockout does not alter microglial phagocytosis of healthy or dead cells
• P2Y_6_R knockout prevents microglial phagocytosis of neurons induced by Aβ *in vivo*
• P2Y_6_R knockout reduces neuronal loss and memory deficits induced by Aβ or Tau in mice

## Graphical abstract

**Figure:**
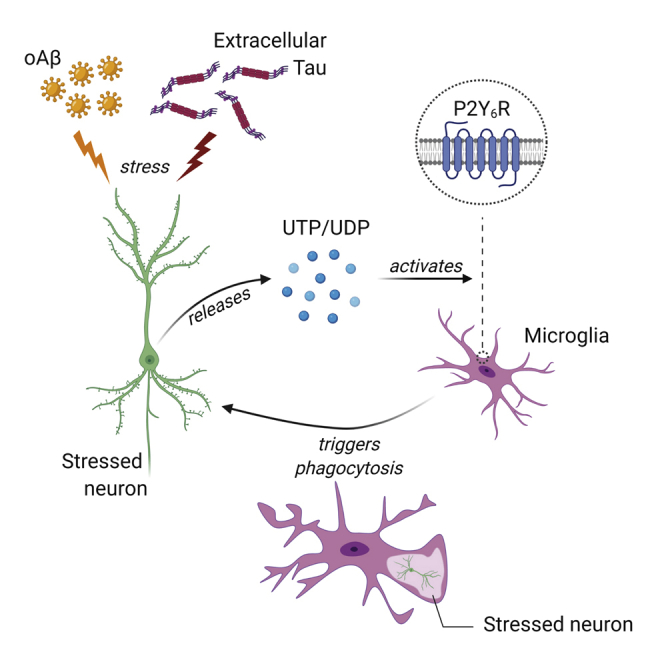

## Teaser

Puigdellívol et al. find that the knockout of the microglial P2Y_6_ receptor, required for microglial engulfment of neurons, prevents neuronal and memory loss in two different mouse models of neurodegeneration, suggesting that neuronal loss in neurodegeneration is due to microglia eating neurons, which may be prevented by blocking the P2Y_6_ receptor.

## Introduction

There is growing evidence that excessive phagocytosis of neurons and/or neuronal parts by microglia may contribute to the brain pathology of neurodegenerative diseases, including Alzheimer’s disease (AD) (Hong et al., 2016; Paolicelli et al., 2017; Butler et al., 2021), as well as aging (Shi et al., 2015; Linnartz-Gerlach et al., 2019). However, microglial phagocytosis of neurons, and thus neuronal cell bodies, is less well-established than phagocytosis of synapses, despite evidence that: microglia make more contact with cell bodies (Cserép et al., 2020), microglia remove neurons or neuronal precursors during development (Cunningham et al., 2013; Anderson et al., 2019), and microglia phagocytose stressed neurons after ischemia (Neher et al., 2013; Alawieh et al., 2018). Importantly, phagocytosis of live cells results in death of the engulfed cells, a type of cell death termed phagoptosis, i.e., cell death by phagocytosis (Brown and Neher, 2014). We and others have shown that microglia can cause neuronal loss and death by phagocytosis of stressed-but-viable neurons in some conditions (Brown and Neher, 2014; Neher et al., 2011; Brelstaff et al., 2018), but whether this contributes to neurodegeneration is unknown (Fricker et al., 2018). Neuronal loss occurs relatively late in AD but correlates well with dementia (Andrade-Moraes et al., 2013). This suggests the possibility that blocking such neuronal loss (for example, by blocking microglial phagocytosis of stressed neurons) may stop or delay disease progression, even after diagnosis.

However, non-specific inhibition of microglial phagocytosis may be detrimental by blocking phagocytosis of dead cells, debris, protein aggregates, and/or pathogens. Indeed, most phagocytic receptors and opsonins recognize dead cells, debris, protein aggregates, and/or pathogens, so blocking such phagocytic receptors may be deleterious (Tay et al., 2018; Vilalta and Brown, 2018; Salter and Stevens, 2017; Gabandé-Rodriguez et al., 2020). However, the microglial P2Y_6_ receptor (P2Y_6_R) is potentially a good target because it is activated by extracellular uridine diphosphate (UDP) released by stressed or damaged neurons (Koizumi et al., 2007), whereas dead cells, debris, or protein aggregates cannot release UDP, and therefore they are unlikely to be recognized by this receptor. P2Y_6_R is a G-protein-coupled receptor, encoded by the *P2ry6* gene, and within the brain is almost exclusively expressed by microglia (Koizumi et al., 2007; Moore et al., 2001; Spangenberg et al., 2019). Microglial P2Y_6_R has been shown to mediate microglial phagocytosis *in vivo* and *in vitro*, and stressed/damaged neurons were shown to release uridine triphosphate (UTP) and UDP, which in turn activates P2Y_6_R on microglia, triggering the formation of the phagocytic cup (Koizumi et al., 2007). We previously found that an inhibitor of P2Y_6_R, N,N’’-1,4-Butanediylbis[N’-(3-isothiocyanatophenyl)thiourea (MRS2578), prevented neuronal loss induced by lipopolysaccharide (LPS) injected into brain or cell cultures of wild-type (WT) animals (Neher et al., 2014), but it remains unclear (1) whether this was actually mediated by P2Y_6_R, (2) whether P2Y_6_R inhibition is beneficial or detrimental, (3) whether P2Y_6_R mediates the phagocytosis of alive or dead cells/targets, and (4) whether neurodegeneration is mediated by P2Y_6_R-activated microglial phagocytosis. The work described here seeks to determine whether microglial phagocytosis contributes to neurodegeneration and whether this can be prevented by blocking the microglial P2Y_6_ receptor.

## Results

### *P2ry6* knockout in mice prevents Aβ-induced microglial phagocytosis of neurons, neuronal loss, and memory deficits *in vivo*

**Figure 1:**
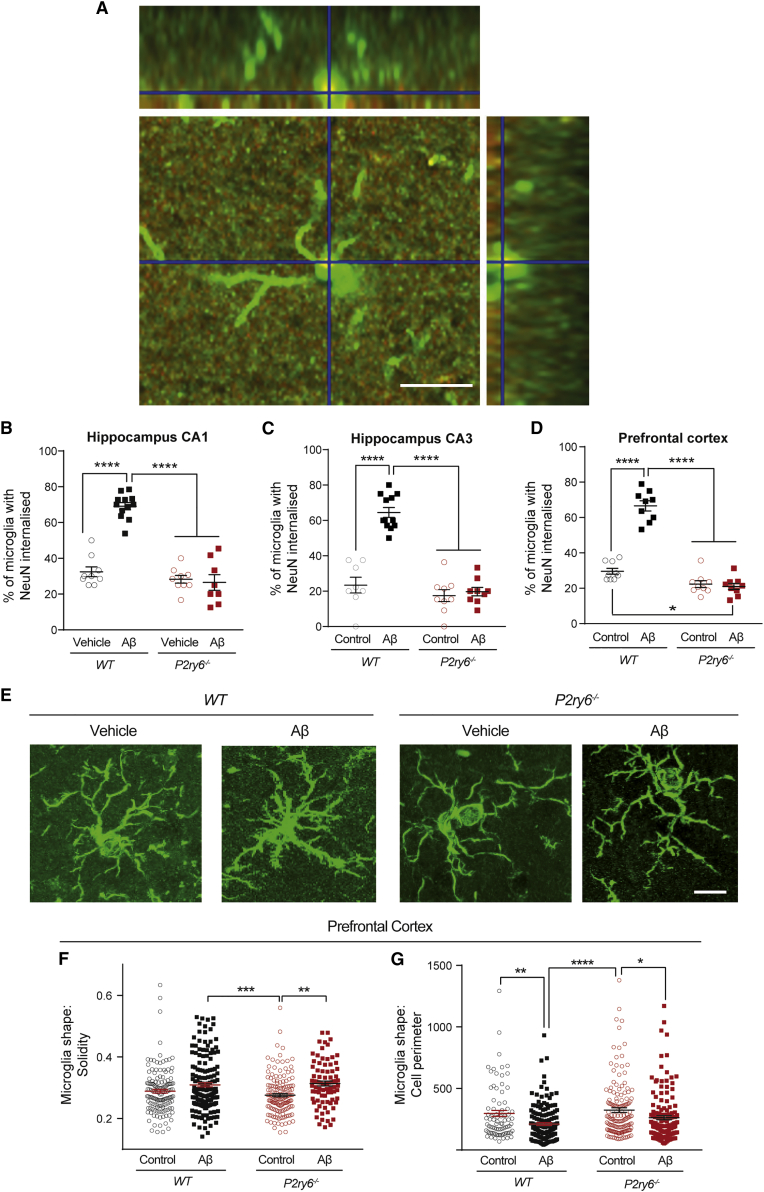
Aβ injection induces microglial phagocytosis of neurons in wild-type but not *P2ry6* knockout mice Analysis of microglial phagocytosis of neuronal material by manual quantification of the percentage of Iba1-positive microglia with NeuN-positive material ingested in matched sections of hippocampus and prefrontal cortex following i.c.v. injection of Aβ or PBS (control) in wild-type (WT) and *P2ry6* knockout (*P2ry6*^−/−^) mice. (A) Representative X-Y, X-Z, and Y-Z projection of an Iba1-positive (green) microglial cell with NeuN-positive material (red) inside (overlap yellow). Scale bar: 10 μm. (B–D) Percentage of microglia with NeuN material internalized in CA1 (B), CA3 (C), and prefrontal cortex (D). (E) Representative image of Iba1^+^ microglia in the hippocampal CA1 area. Scale bar, 10 μm. (B–G) Automated quantification of microglia shape solidity and perimeter in the prefrontal cortex (F and G, respectively). Each data point represents one field of view (A–D) or one Iba1^+^ cell (F and G). Number of mice: WT-Aβ = 6, WT+Aβ = 6, KO-Aβ = 4, and KO+Aβ = 4. Error bars indicate mean ± SEM. Data were analyzed by two-way ANOVA with Tukey-corrected post hoc comparisons. ^∗^p < 0.05, ^∗∗^p < 0.01, ^∗∗∗^p < 0.001, and ^∗∗∗∗^p < 0.0001. For each graph, all genotypes were compared, and if there is no marker of significance on the graph, then any difference was not significant. See also Figures S1–S3.

To determine whether P2Y_6_R is involved in neurodegeneration, we used an acute amyloid model of AD (Prediger et al., 2007), known to feature excessive microglial phagocytosis (Hong et al., 2016). We stereotactically injected 400 pmol of aggregated amyloid beta (Aβ) into the right lateral ventricle of WT and *P2ry6*^*−/−*^ mice, and three days later we measured microglial phagocytosis of neurons, quantified as the number of microglia containing neuronal nuclear (NeuN-positive) material in brain sections. Strikingly, we found that Aβ induced a three-fold increase in microglial phagocytosis of neurons in WT mice in all brain areas analyzed (Figures 1A–1D and S1). In contrast, Aβ injection induced no increase in NeuN-positive material inside microglia in *P2ry6*^*−/−*^ mice (Figures 1A–1D). These findings indicate that Aβ injection induces microglial phagocytosis of neurons *in vivo* and that blocking P2Y_6_R is sufficient to prevent this.

To investigate whether P2Y_6_R affects microglial activation, we analyzed microglial density and morphology in the hippocampus and prefrontal cortex of WT and *P2ry6*^*−/−*^ mice. Microglial density was not significantly altered by Aβ treatment in WT or *P2ry6*^*−/−*^ mice, except in hippocampal area CA1, and P2Y_6_R knockout did not reduce microglial density in any area or condition (Figures S2A–S2C), indicating that any neuroprotection in *P2ry6*^*−/−*^ mice is not due to having fewer microglia or less microglial proliferation. Aβ caused a mild, morphological activation of microglia (measured as increased microglial solidity and decreased perimeter), which was reduced by P2ry6^−/−^ in CA1 but unaffected in CA3 and prefrontal cortex (Figures 1E–1G, S1O–S1R, and S2). Thus, *P2ry6* knockout has little effect on Aβ-induced microglial activation as measured by microglial proliferation and morphology *in vivo*, consistent with our previous findings that P2Y_6_R inhibition has no effect on microglial activation as measured by release of cytokines and nitric oxide or isolectin B4 binding *in vivo* (Neher et al., 2014). To analyze this in more detail, we measured inflammatory cytokine release by cultured primary microglia from WT and *P2ry6* knockout mice, treated ± lipopolysaccharide, using an ELISA array of 62 cytokines and chemokines. There were no significant differences in the LPS-induced fold change in release of any cytokine/chemokine between *P2ry6*^*+/+*^ and *P2ry6*^*−/−*^ microglia (Figure S3). These results confirm that *P2ry6* knockout had little effect on microglial activation.

**Figure 2:**
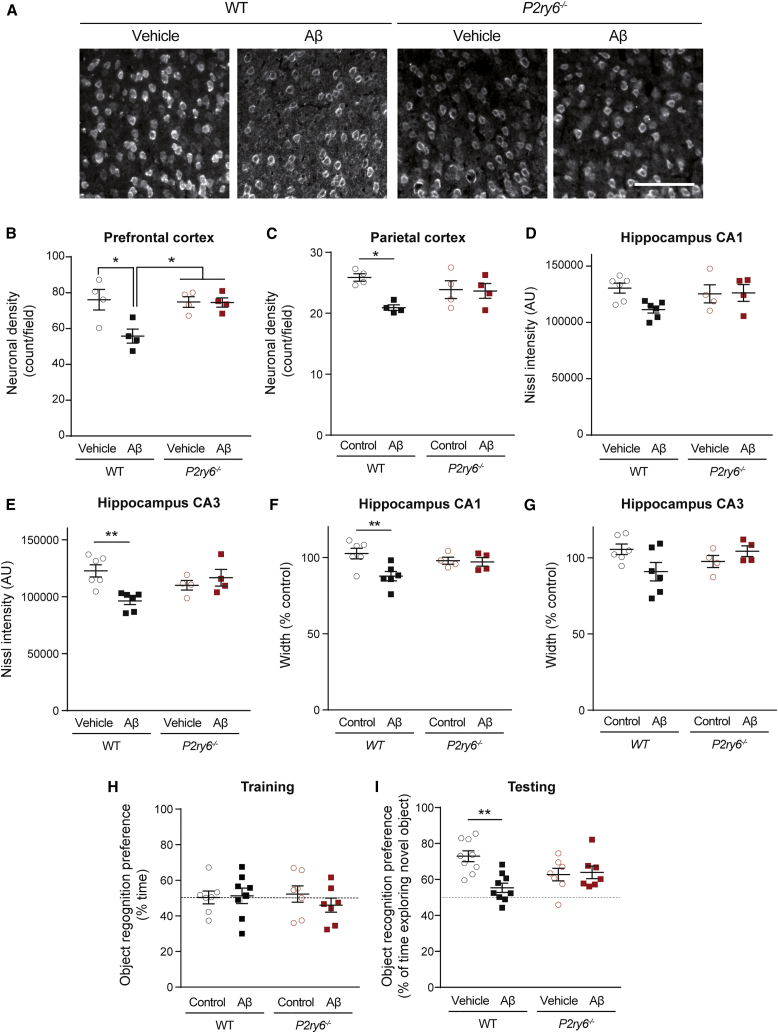
*P2ry6* knockout mice are protected against Aβ-induced neuronal loss and memory deficit (A) Representative images of NeuN-positive staining of prefrontal cortex area 14 days after i.c.v. injection of Aβ1-40 (Aβ) or PBS (control) in WT and *P2ry6* knockout (*P2ry6*^*−/−*^) mice. Scale bar: 100 μm. (B and C) Quantification of NeuN-positive neuron densities of prefrontal cortex (B) and parietal cortex (C) in Aβ-treated and control WT and knockout mice. (D and E) Quantification showing average Nissl intensity of CA1 (D) and CA3 (E) area per animal after background correction. (F and G) Average width of CA1 (F) and CA3 (G) area per animal, normalized to average WT control (100%). WT and *P2ry6*^*−/−*^ mice were tested for novel-object recognition 14 days after i.c.v. injection of Aβ1-40 (Aβ) or PBS (control). (H) Percentage of time each animal spent exploring two identical objects during the training session of the NORT. (I) Novel-object recognition, 2-min retention interval. Dashed lines indicate a 50% chance level. Each data point represents one animal. Error bars indicate mean ± SEM. Data were analyzed by two-way ANOVA with Tukey-corrected post hoc comparisons. ^∗^p < 0.05 and ^∗∗^p < 0.01. For each graph, all genotypes were compared, and if there is no marker of significance on the graph, then any difference was not significant. See also Figure S4.

To determine whether microglial phagocytosis of neurons was associated with subsequent neuronal loss, we measured neuronal densities two weeks after Aβ injection. In WT mice, Aβ injection reduced neuronal density in the prefrontal cortex (Figures 2A, 2B, S4A, and S4D) and median and lateral parietal association cortex (Figure 2C). By contrast, Aβ caused no significant loss of cortical neurons in *P2ry6*^*−/−*^ mice (Figures 2A–2C). Similarly, Aβ injection reduced the density and thickness of the CA1 and CA3 subfields of the hippocampus of WT mice, but not *P2ry6*^*−/−*^ mice (Figures 2D–2G, S4A–S4C, and S4E). Altogether, these results indicate that Aβ-induced neuronal loss is mediated by P2Y_6_R.

Finally, because blocking microglial phagocytosis of neurons could be beneficial or detrimental for brain function, we tested whether blocking P2Y_6_R affected Aβ-induced memory deficits by evaluating novel-object recognition in the mice (Figures 2H and 2I). As expected, Aβ severely impaired novel-object recognition in WT mice (Figure 2I). However, Aβ had no significant effect on novel-object recognition in *P2ry6*^*−/−*^ mice (Figure 2I), indicating that P2Y_6_R is required for the Aβ-induced memory impairment and blocking P2Y_6_R prevents the memory deficit.

Overall, these results indicate that *P2ry6* knockout prevents the microglial phagocytosis of neurons, neuronal loss, and memory deficits induced by Aβ.

### *P2ry6* knockout reduces neuronal loss and prevents memory deficits in a P301S TAU mouse model of neurodegeneration

The above model of neurodegeneration is acute and amyloid-induced, whereas AD is chronic and appears to require tau pathology, which can be driven by pathways independent of Aβ (van der Kant et al., 2020), so we next tested whether *P2ry6* knockout is beneficial in a chronic model of tauopathy. To do this, we crossed *P2ry6*^*−/−*^ mice with homozygous TgP301S mice expressing human mutant P301S tau specifically in neurons (recently rederived into a C57BL/6J background) (Figure S5A), which develop progressive tau aggregation, neuronal loss, and behavioral impairment (Allen et al., 2002; Hampton et al., 2010).

**Figure 3:**
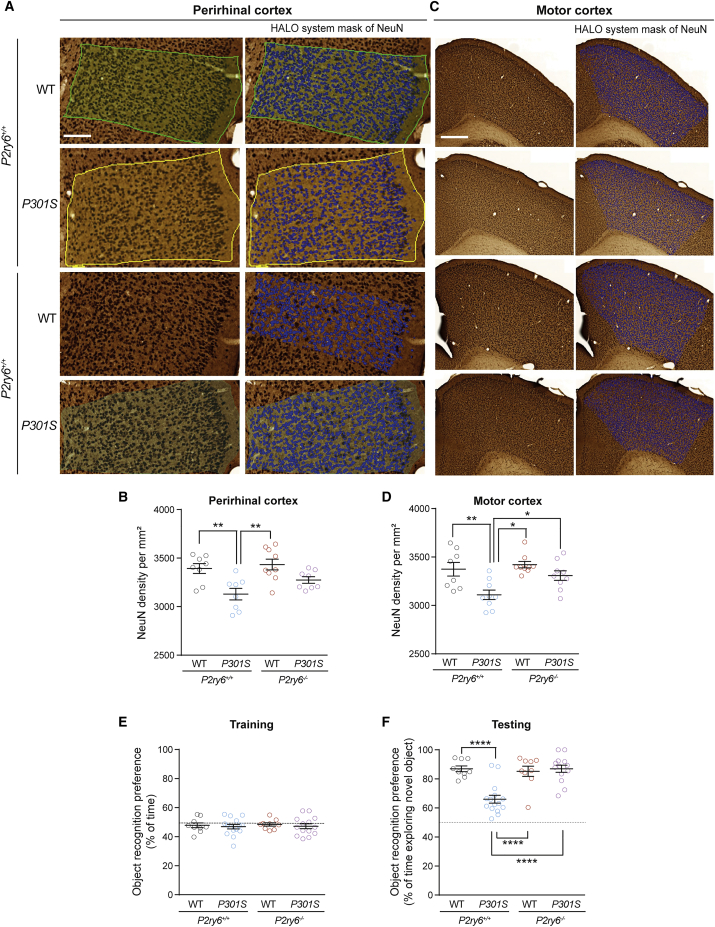
P301S tau mice have cortical neuron loss and memory deficit prevented by crossing with *P2ry6* knockout mice WT and *P2ry6*^*−/−*^ mice were crossed with P301S tau mice, aged, and tested at six months. (A) Representative coronal section of perirhinal cortex immunostained for NeuN and nuclei identified by HALO system. Scale bar: 100μm. (B) Quantification of neuronal density within the perirhinal cortex. (C) Representative coronal section of motor cortex immunostained for NeuN and nuclei identified by HALO system. Scale bar: 500μm. (D) Quantification of neuronal density within the motor cortex. Data = mean ± SEM (5–6 slices/animal, n = 8–9 animals per genotype). Statistical analysis was two-way ANOVA with post hoc Tukey’s multiple comparison test. ^∗^p < 0.05, ^∗∗^p < 0.01, and ^∗∗∗^p < 0.001. At six months of age, mice were tested for novel-object recognition (NORT, testing 24 h after training). (E) Percentage of time each animal spent exploring two identical objects during the training session of the NORT. (F) Novel-object recognition, 24 h after training. Dashed lines indicate a 50% chance level. Each data point represents one animal, and error bars represent mean ± SEM. Data were analyzed by two-way ANOVA with Tukey-corrected post hoc comparisons. ^∗∗∗∗^p < 0.0001. For each graph, all genotypes were compared, and if there is no marker of significance on the graph, then any difference was not significant. See also Figures S5, S6, and S8.

P301S mice lose cortical neurons (Hampton et al., 2010; Yang et al., 2015 & 2017), so we first tested whether genetic ablation of *P2ry6* is sufficient to prevent the neuronal loss observed in the P301S mouse model at seven months of age. We found about 15% neuronal loss in the perirhinal cortex of P301S mice compared to WT mice (Figures 3A and 3B), similar to the loss previously reported (Yang et al., 2015, 2017). However, no significant neuronal loss was found in the perirhinal cortex of double-transgenic (*P2r6y*^*−/−*^:P301S^+/+^) mice compared to WT (*P2ry6*^*+/+*^:P301S^−/−^) mice (Figures 3A and 3B). Similarly, in the motor cortex, P301S mice had a lower neuronal density than WT mice (as previously shown in Hampton et al., 2010), but this neuronal loss was prevented in the double-transgenic mice (Figures 3C and 3D). These data indicated that the lack of *P2ry6* reduced neuronal loss in P301S mice.

Given that neuronal loss in the perirhinal cortex, a brain region critical for object-recognition memory (Bartko et al., 2007; Mumby and Pinel, 1994), was previously found to associate with memory deficits in the P301S model (Yang et al., 2015, 2017), we next examined whether genetic ablation of *P2ry6* is sufficient to prevent memory deficits in P301S mice. After confirming that there were no significant differences between genotypes at six months of age in body weight (Figure S5B), spontaneous locomotion (Figure S5C), anxiety-like behavior (Figures S5D–S5I), or exploration of objects during the training phase of the object-recognition test (Figure 3E), we next assessed memory performance by novel-object recognition 24 h after training. As expected, we found that P301S (*P2ry6*^*+/+*^:P301S^+/+^) mice had substantial deficits in recognition memory compared to WT (*P2ry6*^*+/+*^:P301S^−/−^) mice (Figure 3F). However, double-transgenic mice (*P2ry6*^*−/−*^:P301S^+/+^), expressing human mutant P301S tau but lacking *P2ry6* expression, had no memory deficit compared to WT mice and substantially better memory than P301S mice (Figure 3F), indicating that *P2ry6* knockout prevented tau-induced memory deficits.

Given that our data demonstrate that genetic ablation of *P2ry6* in homozygous TgP301S tau mice fully prevented the manifestation of memory deficits, we next explored whether the lack of *P2ry6* could improve the severe spinal cord pathology exhibited by TgP301S mice (Allen et al., 2002). Thus, motor coordination, hindlimb clasping, gait, and kyphosis were examined at seven months of age, just before sacrificing the animals. We found that double-transgenic (*P2ry6*^*−/−*^:P301S^+/+^) mice had the same severe spinal cord pathology as P301S (*P2ry6*^*+/+*^:P301S^+/+^) mice (Figure S6), which suggests a different mechanism or severity of neurodegeneration in the spinal cord from the brain in this model of tauopathy.

### *P2ry6* knockout prevents neuronal loss induced by tau, Aβ, and UDP in glial-neuronal cultures

**Figure 4:**
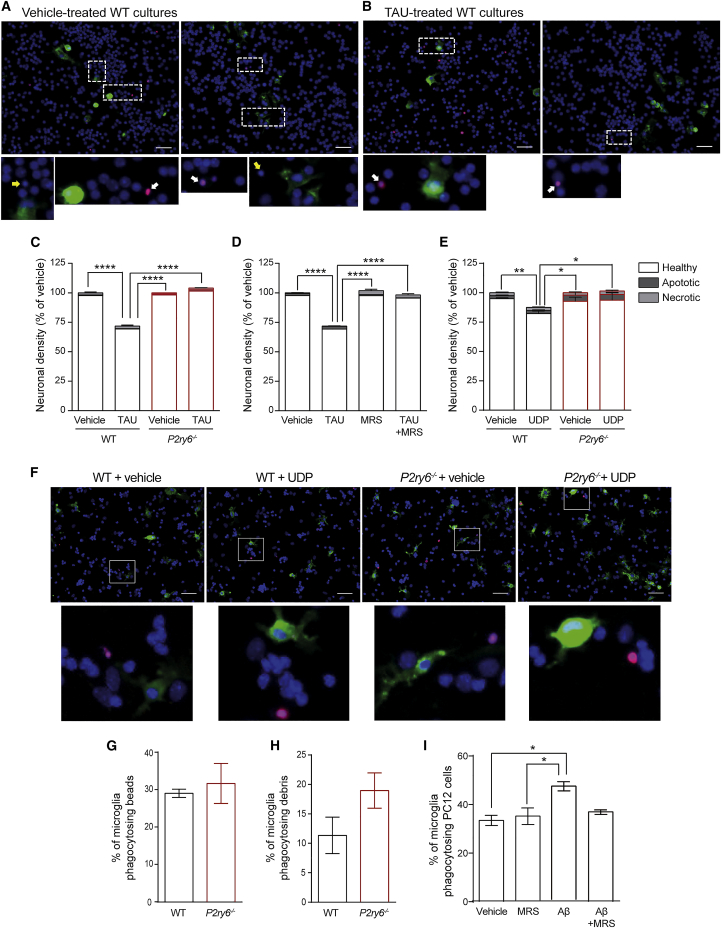
*P2ry6* knockout protects from neuronal loss induced by TAU and UDP in glial/neuronal cultures and reduces microglial phagocytosis of stressed cells, but not phagocytosis of beads or debris (A–F) Mixed neuronal-glial cultures from cerebella of WT or *P2ry6* knockout (*P2ry6*^*−/−*^) mice were treated for 3 days with ± 3 μM tau (A and C), 3 μM tau ± 1 μM MRS2578 (D), or 100 μM UDP (E and F), then density of necrotic, apoptotic, and healthy neurons was counted. Scale bar: 50 microns. (A and F) Representative images (upper panels) and corresponding insets (bottom panels) showing microglia (IB4, green), PI (as a necrotic marker, red), and nuclei (Hoechst, blue) staining in (A) mixed neuronal-glial cerebellar cultures from WT mice treated ± 3 μM tau, or (F) mixed neuronal-glial cerebellar cultures from WT and *P2ry6*^*−/−*^ treated ± 100 μM UDP. Apoptotic neurons (yellow arrows) can be seen as smaller Hoechst-positive nuclei (due to nuclear condensation), and necrotic neurons (white arrows) are stained with PI (red). Notice that apoptotic and necrotic neurons are very rare in these conditions. Data = mean ± SEM (N = 3 independent experiments; each experiment in triplicate). Data were analyzed by two-way ANOVA with Tukey-corrected post hoc comparisons. ^∗^p < 0.05 ^∗∗^p < 0.01, and ^∗∗∗∗^p < 0.0001. (G and H) Percentage of microglia isolated from WT or *P2ry6*^*−/−*^ mice phagocytosing 5-micron beads (G) and neuronal debris (H). (I) Percentage of microglia phagocytosing PC12 cells ± 500 nM Aβ ± 1 μM MRS2578. n = 3–5 independent experiments for each. Data = mean ± SEM. Data were analyzed by two-way ANOVA with post hoc Tukey’s multiple comparison test (I) and two-sample t test (G and H). ^∗^p < 0.05. For each graph, all treatments/genotypes were compared, and if there is no marker of significance on the graph, then any difference was not significant. See also Figure S7.

To further investigate the underlying mechanisms by which P2Y_6_R is involved in neuronal loss, we used glial-neuronal cultures, isolated from brains of WT and P2Y_6_R knockout (*P2ry6*^*−/−*^) mice, and treated with tau or Aβ. Both tau and Aβ accumulate in the brains of individuals affected by AD and other neurodegenerative diseases (Chi et al., 2018; Sebastián-Serrano et al., 2018; Gallardo and Holtzman 2019). We have previously shown that tau and Aβ induce neuronal loss in glial-neuronal cultures via microglial phagocytosis (Neher et al., 2011; Brelstaff et al., 2018; Pampuscenko et al., 2020, 2021), so we tested here whether this neuronal loss was mediated by P2Y_6_R. We found that the addition of extracellular tau protein (2N4R isoform) caused neuronal loss without inducing apoptosis or necrosis in mixed glial-neuronal cultures from WT mice (Figures 4A–4D). However, tau induced no significant neuronal loss in cultures from P2Y_6_R knockout (*P2ry6*^*−/−*^) mice (Figure 4C). Similarly, 1 μM MRS2578 (a P2Y_6_R inhibitor) prevented tau-induced neuronal loss in WT cultures (Figure 4D), indicating that pharmacological inhibition of P2Y_6_R is as protective as *P2ry6* knockout. Aβ also induced neuronal loss in glial-neuronal cultures from WT mice, as previously reported (Yang et al., 2015), but this loss was reduced in glial-neuronal cultures from *P2ry6*^*−/−*^ mice (Figure S7A).

P2Y_6_R is sensitively and selectively activated by extracellular UDP, so we tested whether UDP alone was sufficient to induce neuronal loss via P2Y_6_R. Addition of UDP to mixed glial-neuronal cultures from WT mice induced neuronal loss without any increase in neuronal apoptosis or necrosis (Figures 4E and 4F). However, UDP induced no neuronal loss in cultures from *P2ry6*^*−/−*^ mice (Figures 4E and 4F). Thus, UDP activation of microglial P2Y_6_R is sufficient to induce neuronal loss, and knockout of the UDP receptor P2Y_6_R is sufficient to prevent neuronal loss induced by UDP, Aβ, and tau.

### UDP/P2Y_6_R mediates phagocytosis of stressed-but-viable cells, but not debris or dead cells, *in vitro*

Our data indicate that blocking P2Y_6_R prevents neuronal loss induced by Tau and Aβ both *in vitro* and *in vivo*, potentially by blocking microglial phagocytosis. However, it is unclear under what conditions and what targets may be phagocytosed via P2Y_6_R. To investigate this, we first compared phagocytosis of different targets by primary microglia from WT and *P2ry6*^*−/−*^ mice and found that phagocytosis of beads (Figure 4G) and neuronal debris (Figure 4H) was not significantly different between the two genotypes.

We next used a model system of BV2 microglial cells phagocytosing PC12 (neuroendocrine) cells. UDP release from the PC12 cells was bioassayed using astrocytoma cells stably transfected with *P2ry6*. Stressing the PC12 cells by treatment with 250 nM Aβ increased extracellular UDP levels (from 38 ± 13 nM to 326 ± 71 nM, p = 0.007), without inducing any PC12 cell death (Figure S7B). Importantly, when such Aβ-stressed PC12 cells were incubated with BV2 cells, there was an increase in microglial phagocytosis of the PC12 cells that was prevented by inhibiting P2Y_6_R with MRS2578, whereas MRS2578 had no significant effect on the phagocytosis of untreated/unstressed PC12 cells (Figure S7C). In contrast, BV2 phagocytosis of necrotic PC12 cells (modeling “dead” cells), was unaffected by inhibiting P2Y_6_R with MRS2578 (Figure S7D). We repeated some of these experiments with primary microglia from WT mice (rather than BV-2 microglia) and found that inhibition of P2Y_6_R with MRS2578 prevented microglial phagocytosis of PC12 cells stressed with 250 nM Aβ, but MRS2578 did not inhibit phagocytosis of unstressed PC12 cells (Figure 4I). Thus, our data indicate that P2Y_6_R mediates the phagocytosis of stressed-but-viable cells, but not the phagocytosis of healthy cells, dead cells, cellular debris, or inert beads. This is consistent with UDP being released from stressed cells (Koizumi et al., 2007; Giuliani et al., 2019), but not from necrotic cells or debris, as these contain no UDP because their membranes are ruptured.

## Discussion

Neuronal loss occurs mainly after diagnosis of Alzheimer’s disease and correlates with dementia symptoms (Andrade-Moraes et al., 2013). Thus, it is possible that dementia progression may be stopped after AD diagnosis by blocking such neuronal loss. In this study, we found that *P2ry6* knockout prevented neuronal loss induced by UDP, Tau, and Aβ in glial-neuronal cultures. We have previously shown that this neuronal loss induced by UDP, Tau, and Aβ is mediated by microglial phagocytosis of stressed-but-viable neurons (Neher et al., 2011 & 2014; Brelstaff et al., 2018; Pampuscenko et al., 2020), and the results here indicate this is mediated by the engulfment receptor P2Y_6_R. In addition, we show here that blocking P2Y_6_R does not inactivate phagocytosis generally (there is no inhibition of phagocytosis of beads or neuronal debris) but does reduce phagocytosis of stressed cells. One explanation of this specificity is that stressed cells contain UDP that can be released to activate P2Y_6_R (Koizumi et al., 2007), whereas beads and debris (with ruptured membranes) contain no UDP and therefore cannot activate P2Y_6_R to induce engulfment. Most phagocytic receptors, such as Mer tyrosine kinase, Axl, TyroB, triggering receptor expressed on myeloid cells 2 (TREM2), TIM4, BAI1, complement receptor 3, and the vitronectin receptor, recognize dead or dying cells, debris, protein aggregates, and/or pathogens, so that chronic inhibition can be detrimental (Cserép et al., 2020; Tay et al., 2018; Vilalta and Brown 2018; Salter and Stevens 2017). As P2Y_6_R is specifically activated by UDP, it may help discriminate stressed-but-viable cells from dead cells and debris and thus may be a better treatment target to block excessive microglial phagocytosis.

To test whether P2Y_6_R inactivation is beneficial in a chronic model of neurodegeneration we used P301S TAU mice, which develop tauopathy, neuronal loss, and memory loss (Allen et al., 2002). We crossed *P2ry6*^*−/−*^ mice with P301S TAU mice and found that P2Y_6_R inactivation partially prevented the tau-induced memory and neuronal loss. Note, however, that the severe spinal cord pathology present in P301S TAU knockin mice was not ameliorated by inactivation of P2Y_6_R, indicating that the aggressive spinal cord pathology in this model is not mediated by P2Y_6_R. Nevertheless, the prevention of brain pathology by *P2ry6* knockout at a late stage in this model is impressive, indicating an important role for P2Y_6_R in the observed neurodegeneration. This is consistent with culture experiments (Brelstaff et al., 2018) showing that neurons with TAU filaments (cultured from P301S TAU mice) were preferentially phagocytosed by isolated microglia, resulting in neuronal death by phagocytosis; i.e., the neurons with TAU aggregates died by phagoptosis. Moreover, given the absence of apoptosis or necrosis in the P301S TAU knockin mice (Allen et al., 2002), our results suggest that phagoptosis (i.e., cell death by phagocytosis) may be a key mechanism of brain neuronal death in this model of chronic neurodegeneration.

As *P2ry6* knockout prevented Aβ-induced neuronal loss *in vitro*, and the Aβ injection *in vivo* model is known to feature excessive microglial phagocytosis, we tested whether *P2ry6* knockout affected Aβ-induced neuronal loss in this model and whether microglial phagocytosis of neuronal material contributes to such loss. We found that WT mice injected with Aβ lost neurons in the hippocampus and cortex and performed significantly worse in a learning and memory task (novel-object recognition), consistent with previous studies using the same model (Prediger et al., 2007). Our novel observation that *P2ry6*^*−/−*^ mice were protected against the memory impairment and neuronal loss induced by Aβ suggests that P2Y_6_R signaling may be involved in the neuronal loss associated with the amyloid pathology of AD. Importantly, we found that Aβ induced a large increase in the proportion of microglia containing NeuN^+^ neuronal material, and this increase was entirely prevented in *P2ry6*^*−/−*^ mice. It is possible, but unlikely, that NeuN^+^ puncta enter microglia by processes other than phagocytosis at the time that NeuN^+^ neurons are lost. Thus, Aβ appeared to induce microglial phagocytosis of neurons, and this was prevented by *P2ry6* knockout.

Interestingly, *P2ry6* knockout did not prevent the morphological activation of microglia after Aβ treatment *in vivo* and had minimal effects on cytokine and chemokine release *in vitro*, consistent with the previous findings by us and others that microglial activation and cytokine release is not affected by inhibition of P2Y_6_R (Neher et al., 2014; Wen et al., 2020). Together, these findings suggest that *P2ry6* knockout prevents Aβ-induced neuronal loss by preventing microglial phagocytosis of neurons rather than by preventing microglial activation itself. Although we lack *in vivo* evidence of microglial phagocytosis of specifically live neurons, which is not currently possible to image *in vivo*, our results suggest that, in WT mice, Aβ treatment causes microglia to phagocytose otherwise viable neurons, as *P2ry6* knockout prevents the observed increase in microglial phagocytosis of neurons three days after Aβ treatment, resulting in viable neurons left behind two weeks later and improved brain function as measured by novel object recognition. This suggests that in WT animals, the neurons were alive when phagocytosed by microglia. UDP is released from stressed neurons and activates formation of the phagocytic cup, driving engulfment (Koizumi et al., 2007). Importantly, this could mean that P2Y_6_R is part of a final common pathway of neuronal engulfment under multiple inflammatory conditions.

In the mouse brain, the P2Y_6_ receptor is mainly, but not exclusively, expressed in microglia (Moore et al., 2001; Spangenberg et al., 2019); its expression has also been found in neurons of the arcuate nucleus of the hypothalamus (Steculorum et al., 2015), in peripheral macrophages (Garcia et al., 2014), and dendritic cells (Li et al., 2020). Thus, we cannot rule out that some of the effects observed in our study partially involved the role of P2Y_6_R on these other cell populations. However, quantitatively this contribution is likely to be small. Similarly, we cannot rule out that the P2Y_6_ receptor is involved in regulating other cellular processes, such as autophagy, but P2Y_6_R is not known to regulate any relevant process other than phagocytosis, and its role in microglial phagocytosis appears sufficient to explain the effects seen here.

We conclude that the P2Y_6_ receptor contributes to neurodegeneration induced by Aβ and TAU at least in part by microglial phagocytosis of neurons. Overall, our findings suggest a model in which UDP released from stressed-but-viable neurons contributes to neuronal loss under inflammatory conditions by promoting phagocytosis of otherwise viable neurons via P2Y_6_R (see graphical abstract, created with BioRender, for proposed model). The finding that neuronal death during neurodegeneration is at least partially mediated by microglial phagocytosis provides new perspectives on the nature of neurodegeneration and how to prevent it. In particular, as the neurodegeneration of AD is thought to be induced by Aβ and TAU, and *P2ry6* knockout prevented neuronal loss induced by these factors in mice, P2Y_6_R inhibition might be beneficial in Alzheimer’s patients. Thus, our study encourages the development of safe and clinically applicable P2Y_6_R antagonists to block microglial phagocytosis of stressed-but-viable neurons, which might prevent memory deficits and neuronal loss in neurodegenerative diseases and other brain pathologies.

### Limitations of the study

This paper has the following general limitations: the experiments were done in mice, mouse cells, and mouse models of human disease, so we do not know that the results will extrapolate to humans and human disease. The *P2ry6* knockout was in all cells, rather than microglia specifically, so we do not know that the effects were due to microglia exclusively. *P2ry6* knockout might affect unknown processes (other than microglial phagocytosis) that impact pathology. We have not directly measured microglial phagocytosis of neurons or determined whether neurons are phagocytosed alive by microglia. Examining additional phagocytic markers would have been useful to determine whether the neuronal nuclei within microglia colocalized with lysosomes.

## STAR★Methods

### Key resources table

**Table:** 

REAGENT or RESOURCE	SOURCE	IDENTIFIER
**Antibodies**		

Mouse monoclonal Anti-NeuN Antibody, clone A60	Millipore	Cat #Mab377; RRID: AB_2298772
Rabbit polyclonal anti-Iba1	Wako	Cat #019-19741; RRID: AB_839504
Goat anti-mouse biotinylated secondary antibody	Vector Laboratories	Cat #BA-9200; RRID: AB_2336171

**Chemicals, peptides, and recombinant proteins**		

Hoechst 33342	Sigma-Aldrich	Cat #14533
Isolectin-B4-AlexaFluor 488 from *Griffonia simplicifolia* (IB4)	ThermoFisher	Cat #I21411; RRID: AB_2314662
Propidium iodide	Sigma-Aldrich	Cat #P4170
Synthetic human amyloid β 1-40 peptide	Bachem	Cat #H11940500
Monomeric amyloid β1-42 peptide	Anaspec	Cat #AS20276
Cytochalasin D	Sigma-Aldrich	Cat #C8273
Uridine 5’-diphosphate disodium salt hydrate	Sigma-Aldrich	Cat #94330
Lipopolysaccharide from *Salmonella enterica* serotype typhimurium	Sigma-Aldrich	Cat #L6143
5-(and-6)-carboxytetramethylrhodamine succinimidyl ester (5(6)-TAMRA SE	Biotium Inc	Cat #BT-90022
MRS2578	Sigma-Aldrich	Cat #711019-86-2
Staurosporin	Sigma-Aldrich	Cat #62966741
Recombinant human Tau protein (isoform 2N4R)	Dr. Vilmante Borutaite (University of Vilnius); PMID: 31834946	N/A
Neuronal debris	This paper	N/A
carboxylated 5-micron beads coupled to fluorescent nile red dye	Spherotech	Cat #FH50562

**Critical commercial assays**		

ABC Elite kit mix (Vectastain ABC Kit (Standard))	Vector Laboratories	Cat #PK-6100
DAB Peroxidase Substrate	Vector Laboratories	Cat #SK-4100
ELISA cytokines and chemokines	Abcam	Cat #ab133995

**Experimental models: Cell lines**		

Human astrocytoma cell line 1321N1	A gift from the Department of Physiology, Development and Neuroscience, University of Cambridge; PMID: 4313504	N/A
Human astrocytoma cell line 1321N1 expressing mCherry	This paper	N/A
Human astrocytoma cell line 1321N1 expressing murine recombinant P2Y_6_ receptor C-terminally tagged with mCherry	This paper	N/A
V-raf/v-myc immortalized murine microglial BV2 cell line	ECACC; PMID: 1578513	RRID: CVCL_0182
Rat pheochromocytoma PC12 cell line	A gift from Dr. Tony Jackson (Department of Biochemistry, University of Cambridge); PMID: 1065897	N/A

**Experimental models: Organisms/strains**		

Mouse: C57Bl/6	Charles River Laboratories	N/A
Mouse: *P2ry6* knockout (*P2ry6*^*-/-*^) on a C57Bl/6 background	Bernard Robaye (ULB Brussels); PMID: 18523137; and this paper	N/A
Mouse: TgP301S tau mice on a C57Bl/6 background	Dr Michel Goedert (Laboratory of Molecular Biology); PMID: 25483398; and this paper	N/A
Mouse: *P2ry6*^*-/-*^ : P301S^+/+^ mice on a C57Bl/6 background	This paper	N/A
Rat: Wistar	Charles River Laboratories	RRID: RGD_2312511

**Oligonucleotides**		

**Primer sequences: *P2ry6* WT and *P2ry6* knockout** Y601s - reverse primer: 5’- TGGAATTCAGACTGAGGACG	Sigma (Primer sequence details in this paper)	Y601s
**Primer sequence: *P2ry6* WT** Y601as - forward primer: 5’- GGTAGCGCTGGAAGCTAATG	Sigma (Primer sequence details in this paper)	Y601as
**Primer sequence: *P2ry6* knockout** Cpl4s -forward primer: 5’- AGGTGTTGTGACAGAAGTGTG	Sigma (Primer sequence details in this paper)	Cpl4s
**Primer sequence: P301S WT and mutant P301S** Cdown - reverse primer: 5’- GCAGCCTAGCTCAGTATAATG	Sigma (Primer sequence details in this paper)	Cdown - reverse primer
**Primer sequence: P301S WT** Nup - forward primer: 5’- CTCCAGATTTGTGTAGAATGGC	Sigma (Primer sequence details in this paper)	Nup - forward primer
**Primer sequences: mutant P301S band:** CT3- forward primer: 5’- CACCCACTCGTTCACTGTCC.	Sigma (Primer sequence details in this paper)	CT3- forward primer

**Software and algorithms**		

ImageJ	https://imagej.nih.gov/ij/	RRID:SCR_003070
SMART junior Panlab	Home (panlab.com)	RRID:SCR_012154
GraphPad Prism Version 6	https://www.graphpad.com/	RRID:SCR_015807
Halo image analysis platform- indica labs algorithm, with few modifications	https://indicalab.com/halo/; This paper	N/A

### Resource availability

#### Lead contact

Further information and requests for resources and reagents should be directed to and will be fulfilled by the lead contact, Guy Charles Brown (gcb3@ca.ac.uk).

#### Materials availability

The new transgenic mice (lacking P2ry6 and expressing human mutant P301S Tau) generated in this study are no longer available due to animal welfare and licenses. However, the parental strains are listed in the Key Resources Table and the crossing of the mice is described in this paper. The P2Y6R-mCherry plasmids generated in this project are no longer available, but the stable astrocytoma cell lines these plasmids were used to generate, expressing P2Y6R-mCherry and mCherry control, are currently available from the lead contact with a completed Materials Transfer Agreement.

### Experimental model and subject details

#### Mice

All animal work was carried out in accordance with the Animals (Scientific Procedures) Act 1986 Amendment Regulations 2012 following ethical review by the University of Cambridge Animal Welfare and Ethical Review Body (AWERB). *P2ry6* knockout (*P2ry6*^*−/−*^) mice were kindly provided by Bernard Robaye (ULB Brussels) and maintained on a C57BL/6 background (Charles River Laboratories). *P2ry6*^*−/−*^ mice and wild-type (WT) littermates were used to establish homozygous WT and *P2ry6*^*−/−*^ sub-lines. In offspring from these sub-lines, littermates were randomly assigned to control and Aβ treatment groups. Details of experimental animals used for Aβ injection studies are given below:

**Table:** 

Study	Treatment group	Genotype	Number of animals	Sex	Age range (weeks)	Weight range at start of procedure (g, grams)
intracerebroventricular injection (i.c.v.) injection of Aβ1-40 (14-day follow-up)	Control	WT	6	female	42 – 46	30-39
	Treatment	WT	6	female	42 – 46	29-40
	Control	*P2ry6*^*−/−*^	4	female	41 – 43	28-40
	Treatment	*P2ry6*^*−/−*^	4	female	41 – 43	30-38
i.c.v. injection of Aβ1-40 (3-day follow-up)	Control	WT	3	female	43 – 49	31-42
	Treatment	WT	4	female	43 – 49	30-38
	Control	*P2ry6*^*−/−*^	3	female	40 – 45	29-36
	Treatment	*P2ry6*^*−/−*^	3	female	40 – 45	30-38

Transgenic homozygous TgP301S tau mice (P301S^+/+^) expressing human mutant P301S tau under the control of the murine Thy1.2 promoter (Allen et al., 2002) were maintained on a C57BL/6 background (Charles River Laboratories). Both female and male mice were used in the present study. Animals were housed in groups of 3 to 5 with access to food and water *ad libitum* in a colony room kept at 19–22°C and 40%–60% humidity, under a 12-h light/dark cycle in i.v.c cages on wood-chip bedding with paper strip nesting material. Regular monitoring of health status revealed no significant presence of pathogens.

#### Generation of double transgenic **P2ry6**^**−/−**^: P301S^+/+^ mice

Homozygous P301S^+/+^ mice were crossed with homozygous *P2ry6*^*−/−*^ mice to obtain double heterozygous P301S^+/−^: *P2ry6*^*+/−*^ mice. These mice were crossed again with double heterozygous P301S^+/−^: *P2ry6*^*+/−*^ mice to obtain the final genotypes: wild-type (WT), *P2ry6* knockout (*P2ry6*^*−/−*^), homozygous TgP301S tau (P301S^+/+^) and double transgenic (*P2ry6*^*−/−*^: P301S^+/+^) mice. All mice were on a C57BL/6 background (Charles River Laboratories). For more information see Figure S8. Genotypes were determined by PCR analysis. Primers for allele genotyping had the following nucleotide sequence: **A) *P2ry6* PCR: For WT band:** (1) Y601s - reverse primer: 5¢- TGGAATTCAGACTGAGGACG, (2) Y601as - forward primer: 5¢- GGTAGCGCTGGAAGCTAATG; **For P2ry6 knockout band:** (1) Y601s - reverse primer: 5¢- TGGAATTCAGACTGAGGACG, (3) Cpl4s -forward primer: 5¢- AGGTGTTGTGACAGAAGTGTG; **B) P301S PCR: For WT band:** (4) Cdown - reverse primer: 5¢- GCAGCCTAGCTCAGTATAATG, (5) Nup - forward primer: 5¢- CTCCAGATTTGTGTAGAATGGC; **For mutant P301S band:** (4) Cdown - reverse primer: 5¢- GCAGCCTAGCTCAGTATAATG, (6) CT3- forward primer: 5¢- CACCCACTCGTTCACTGTCC.

#### Primary cell cultures

Primary mixed neuronal/glial cultures were prepared from cerebella of postnatal day 3-5 WT and *P2ry6* knockout (*P2ry6*^*−/−*^) mouse pups as previously described (Carrillo-Jimenez et al., 2018). After 7-9 days the culture composition of these cultures was 85 ± 5% neurons, 7 ± 3% astrocytes, and 5 ± 3% microglia. Primary mouse microglial cells were prepared as previously described (Carrillo-Jimenez et al., 2018). Briefly, mixed glial cultures were obtained from the cortex of mouse pups (postnatal day 4-7). Isolated primary microglial cultures were obtained by gently vortexing the mixed glial culture for 1 min to detach microglia and centrifuging the supernatant at 150 g for 7 min with no brake. The microglia were resuspended in medium consisting of one-part conditioned media and two parts fresh DMEM supplemented with 10% FBS. The cells were seeded on poly-L-lysine coated 24-well plate at 1 × 10^5^ cells/well density and incubated overnight before the phagocytosis assay. All tissue culture medium was supplemented with 100 U/mL penicillin and 100 μg/mL streptomycin (Invitrogen). All cells were kept in a humidified incubator at 37°C and 5% CO_2_.

#### BV2 and PC12 cell cultures

The v-raf/v-myc immortalized murine microglial BV2 cell line was maintained in DMEM supplemented with 10% FBS (Invitrogen) in T-75 flasks (Nunc). At confluence, the cells were harvested using 0.05% trypsin/ ethylenediaminetetraacetic acid (EDTA) (Invitrogen) and were seeded in 24-well plates (5 × 10^4^ cells/well, Nunc) in DMEM supplemented with 0.5% FBS. Rat pheochromocytoma PC12 cell line was maintained in RPMI-1640 medium supplemented with 10% horse serum (Invitrogen) and 5% FBS in T-75 flask coated with 0.5 mg/mL collagen type IV (Sigma). At confluence, the cells were harvested using 0.05% trypsin/EDTA and were seeded in 10 cm^2^ dish (3 × 10^6^ cells/dish, Falcon), 6-well plate (1 × 10^6^ cells/well, Nunc), or 24-well plate (2.5 × 10^5^ cells/well, Nunc).

#### Human astrocytoma cell line 1321N1

Human astrocytoma cell line 1321N1 was stably transfected with mCherry alone or murine recombinant P2Y_6_ receptor C-terminally tagged with mCherry. Both cell lines were maintained as with BV2 (plus G418 selection) and seeded in a 96-well plate at 2 × 10^4^ cells/well.

### Method details

#### Primary cell culture experiments

Primary mixed neuronal/glial cultures were treated with either 100 μM UDP, 250 nM of monomeric amyloid β1-42 for three days or 3 μM TAU protein (2N4R isoform, expressed in *E. coli*, provided by V. Smirnovas, and prepared as previously described (Pampuscenko et al., 2020)) for two days. When indicated, cultures were pre-incubated with 1 μM MRS2578 (Sigma-Aldrich) for 30 min. To determine the cell viability after UDP and TAU treatment, mixed neuronal/glial cultures were stained with the nuclear dye Hoechst 33342 (5 μg/mL) to identify healthy and apoptotic nuclei, 488–tagged isolectin-B4 (1 μg/mL) to identify microglia and propidium iodide (PI, 2 μg/mL) to identify necrotic cells, and cells were imaged and analyzed as previously described (Carrillo-Jimenez et al., 2018). To assess cell viability after Aβ treatment, we measured the rate of reduction of 3-(4,5-dimethylthiazol-2-yl)-2,5-diphenyltetrazolium (MTT) to formazan by the cells. Thus, mixed neuronal/glial cultures were incubated with MTT (0.58 mg/mL) for 2 h at 37°C. Afterward, the converted dye was liberated from the cells and solubilized by the addition of dimethyl sulfoxide (DMSO), and the absorbance intensity of λ = 590 nm light was measured.

#### Microglial phagocytosis of beads and neuronal debris

5-micron beads (carboxylated and coupled to fluorescent nile red dye, Spherotech) were added at 0.005% (w/v) to primary mouse microglia for 1 h. Media was aspirated and cells washed several times with cold phosphate-buffered saline (PBS), then lifted by trypsinization and resuspended in PBS, and uptake of beads into cells was assessed by flow cytometry (Accuri C6 BD). The percentage of microglia that had taken up beads was quantified using a microglial gate that excluded free beads, and a gate of microglia that had taken up one or more beads. At least 5,000 cells were analyzed for each treatment in replicate.

Neuronal debris was prepared by replacing the culture medium of a live neuronal-glial culture with PBS, scratching and scraping the cells with a cell scraper, and passing cells 10 times through a 0.4 mm × 13 mm syringe needle. Debris was labeled with 50 μM 5-(and-6)-carboxytetramethylrhodamine succinimidyl ester (5(6)-TAMRA SE, from Biotium Inc BT90022) for 15 min at 37°C, then washing twice with PBS using a 5 kDa spin column (10,000 g for 5 min). 30 μg of neuronal debris (corresponding to about 1 × 10^5^ dead neurons) were added to each well of 1 × 10^5^ microglia in a 24-well plate for 1 h at 37°C. Medium was aspirated and cells washed twice with cold PBS, then lifted by trypsinization and resuspended in PBS, and uptake of debris into microglia was assessed by flow cytometry (Accuri C6 BD). The percentage of microglia that had taken up debris was quantified using a microglial gate that excluded debris, and a gate of microglia that had taken up debris. At least 5,000 cells were analyzed for each treatment in replicate.

#### Cytokine and chemokine release from microglia

Primary microglia were isolated from mixed glial cultures from wild-type and P2ry6−/− mice and treated with ± 100 ng/mL lipopolysaccharide for 16 h, then the extracellular cell supernatant was centrifuged at 10,000 RCF to remove cellular debris. The supernatant was then assayed using an ELISA for 62 mouse cytokines and chemokines as per the manufacturer’s instructions (Abcam, ab133995). Densitometric measurements were quantified using ImageJ, and intensity values normalized between membranes using positive control spots.

#### BV2 and PC12 experiments

BV2 cell phagocytosis of PC12 cells was performed and analyzed as previously described (Fricker et al., 2012). BV2 cells were allowed to adhere for 24 h then LPS activated (100 ng/mL) for a further 24 h. The cells were pre-treated ± MRS2578 (1 μM, 60 min) or cytochalasin D (0.5 μM, 30 min) prior to the co-culture as indicated. PC12 cells in suspension were treated with staurosporine or monomeric amyloid-β 1-42 for 24 h. PC12 cells were harvested, stained with TAMRA for 15 min in PBS, and washed in five times excess volumes of PBS. The untreated and treated PC12 cells were seeded on BV2 cells at 3 × 10^5^ cells/well and the co-culture was incubated for 3 h. BV2 cells were stained with IB4-Alexa488 for 15 min prior to the end of the co-culture period. After 3 h, the cultures were washed 3 times with ice-cold PBS to remove un-phagocytosed PC12 cells, and the remaining cells were harvested by trypsinization, centrifuged at 150 x g for 5 min at 4°C, and resuspended in PBS on ice. FL1 (IB4-Alexa488) and FL3 (TAMRA) fluorescence of harvested cells were measured by BD Accuri C6 flow cytometer. FSC and FL1 fluorescence were used to positively select the BV2 cell population and eliminate PC12 cells. 10,000 BV2 cell events per well, from triplicate per condition, were collected, and the proportion of stained BV2 cells that had shifted into a TAMRA (FL3) gate was measured.

For primary microglial phagocytosis of stressed PC12 cells, PC12 cells were pre-treated for 24 h with 500 nM monomeric amyloid beta 1-42 (Anaspec) or vehicle. PC12 cells were TAMRA stained as previously described, then washed, and any clumps were dissociated by treatment with 50 μM EDTA. Primary rat microglia were seeded at 30,000 cells per well and pre-treated with 1 μM MRS2578 or DMSO vehicle for 1 h prior to the addition of 100,000 TAMRA-stained PC12 cells per well. After 90 min of phagocytosis, cells were detached by trypsinization and microglia stained with Alexa488-conjugated IB4, and phagocytosis quantified by flow cytometry as above.

#### Bioassay of UDP concentrations using P2Y_6_ receptors transfected astrocytoma cells

UDP concentrations outside PC12 cells were estimated by adding the medium to astrocytoma cells transfected with the P2Y_6_ receptor and measuring the induced calcium response relative to known amounts of UDP. As previously indicated, human astrocytoma cell line 1321N1 was stably transfected with mCherry alone or murine recombinant P2Y_6_ receptor C-terminally tagged with mCherry. Both cell lines were maintained as with BV2 (plus G418 selection) and seeded in a 96-well plate at 2 × 10^4^ cells/well, washed with Flex buffer, and loaded with 0.5 μM Fura-2 AM for 1 h (plus 0.5 mg/mL Pluronic F-127), washed and replaced with 100 μL Flex buffer, and transferred into the FlexStation 3 Microplate Reader (Molecular Devices) maintained at 37°C. PC12 cells were treated, washed, and resuspended in 1 mL Flex buffer for 1 h incubation at 37°C, then centrifuged and the conditioned Flex buffer supernatant used to stimulate 1321N1 cells expressing P2Y_6_R-mCherry or mCherry.

#### Intracerebroventricular injection of Aβ1-40

Synthetic human amyloid β 1-40 peptide (Bachem) was dissolved in DMSO (Sigma) to 5 mM, diluted in 1x PBS (LifeTech) to 100 μM, and left to aggregate for 24 h at 4°C with gentle agitation. 400 pmoles (4 μl) of aggregated Aβ1-40 or 4 μl PBS (control) were injected into the right ventricle of adult (9-12-month-old) wildtype or *P2ry6*^*−/−*^ mice using a 26-gauge needle on a stereotaxic frame. Injections were carried out under isoflurane anesthesia with appropriate analgesia and post-op care using a stereotaxic frame (Kopf Instruments). Injection coordinates were antero-posterior (AP) −0.6 mm, medio-lateral (ML) 1.2 mm, dorso-ventral (DV) −2.2 mm from Bregma, flat skull. Mice were allowed to recover, and tissues were collected 3 or 14 days after injection.

#### Novel object recognition test (NORT) and spontaneous locomotor activity

Novel object recognition testing was performed in a 30 × 44 cm arena with opaque sides. To evaluate memory loss in the acute Aβ model, 2 or 12 days after PBS or Aβ1-40 i.c.v. injection, animals were habituated in the arena without any objects present for two 10-min sessions two h apart. The next day, two identical objects were presented for a 10-min familiarization session followed by a 2-min retention interval and a 5-min test session with one familiar and one novel object. The order of testing of mice from different experimental groups was randomized on day 1 and maintained in the same order on day 2. Object interaction times and ratios were extracted from digital recordings of the trials using modified “Autotyping” software.

For the P301S mice, NORT was performed similarly to above, but with a 24 h retention time to test long-term memory (Giralt et al., 2012). Briefly, mice were first habituated to the arena in the absence of objects on two consecutive days (15 min/day), when spontaneous locomotor activity (total distance traveled) and anxiety/motivation (distance traveled in periphery v. center of the open field) was measured. On the third day, two similar objects were presented for 10 min (A and A’ objects). Twenty-four h later, the same animals were retested for 5 min in the arena with a familiar (A) and a new (B) object. The object preference was measured as the time exploring each object x 100/time exploring both objects. Animals were tracked and recorded with SMART Junior software (Panlab). Objects and arena were cleaned thoroughly with 70% ethanol and dried after each trial to eliminate odor cues.

#### Motor coordination

Motor coordination, muscle function, and markers of disease progression were evaluated as described in (Guyenet et al., 2010). Measures included hind limb clasping, ledge test, gait, and kyphosis. A composite phenotypic score was also calculated as in (Guyenet et al., 2010).

#### Transcardial perfusion and tissue sectioning

Mice were given terminal anesthesia (150 μl Euthatal intraperitoneal (i.p.)) and, once unresponsive to pain, perfused transcardially, through a 25-gauge needle, with 20 mL PBS pH 7.4 followed by 60 ml 4% paraformaldehyde (PFA), pH 7.4 using a perfusion pump with a flow rate of 4 mL/min. Following perfusion, brains were removed and post-fixed overnight in the same solution, cryoprotected by immersion in an increased 10%–30% sucrose solution until sectioning. Brain sections were cut to 20 μm thickness using a Compresstome VF-200 vibratome (Precisionary Instruments), collected on Superfrost Plus slides (Thermo Fisher), and dried overnight. Serial coronal sections (25 μm) through the whole brain were collected using a sliding microtome and placed in PBS as free-floating sections.

#### Nissl staining

Matched brain slices were identified based on anatomical landmarks and placed directly into a 1:1 ethanol:chloroform mixture and incubated overnight at room temperature. Following rehydration series from 100% ethanol to water, staining was carried out in 0.1% cresyl violet solution at 37°C for 10 min. Slices were then quickly rinsed in water, de-stained in 95% ethanol for 2-5 min, dehydrated in 100% ethanol for 10 min, cleared in xylene for 10 min and coverslips mounted. Stained slides were imaged on a Leica DMI6000 CS microscope with a 10x, 0.3NA air objective and tile scanning option to assemble the entire brain section.

#### Immunostaining of non-free-floating brain slices

All steps were carried out at room temperature unless indicated otherwise. Brain slices were re-hydrated for 1 h in PBS and heat-mediated antigen retrieval was carried out at 95þC for 20 min in citrate buffer (10 mM sodium citrate, 0.05% Tween 20, pH 6.0). Following washes in PBS (6 × 10 min), slices were permeabilized in PBS with 0.5% Triton X-100 for 10 min followed by 1 h incubation in blocking solution (50% normal goat serum in PBS). Slices were then incubated in primary antibody solution (5% normal goat serum in PBS plus appropriate primary antibody) at 4°C overnight. Following washes in PBS (6 × 10 min), slices were incubated with secondary antibody for 2 h, washed (6 × 10 min, PBS), and mounted using Vectashield mounting medium with DAPI (Vector Laboratories). Primary antibodies used were Anti-NeuN (Millipore, mouse monoclonal, 1:500 dilution) and anti-Iba1 (Wako, rabbit polyclonal, 1:500 dilution). Secondary antibodies were Alexa Fluor 488 anti-mouse, Alexa Fluor 568 anti-rabbit, and Alexa Fluor 633 anti-rabbit (all ThermoFisher, goat, 1:1000 dilution). Imaging was carried out on an Olympus FV1000 upright laser-scanning confocal microscope with a 60x, 1.35NA oil immersion objective using 488, 559, and 635 nm laser lines.

#### Image analysis using ImageJ 1.49 software

All image analysis was carried out using ImageJ 1.49 software and all manual counting and quantification was performed blinded to genotype and treatment condition. Following background removal, the average Nissl intensity of the entire hippocampal cornu ammonis 1 (CA1) and cornu ammonis 3 (CA3) areas was quantified and their width was determined at three fixed points along their length. Four brain sections were analyzed per animal, with both right and left sides of the hippocampus included in the analysis. For quantification of neuronal density in the prefrontal cortex, regions of interest of fixed size were placed randomly in anatomically matched sections and NeuN^+^ cells were counted manually. Three areas were counted on each side of the midline for a total of four sections per animal.

Microglia phagocytosis of neuronal material was quantified in sections stained for NeuN and Iba1. Three images were taken for each area of interest (CA1, CA3, prefrontal cortex) per mouse, and z stacks of all microglia (as identified by positive Iba1 staining) were analyzed for NeuN^+^ inclusions inside the microglia in X, Y, and Z dimensions. In order to be considered positive, inclusions had to appear in at least two subsequent z slices. In the same images, microglial shape descriptors were analyzed as described in (Zanier et al., 2015). Following automated background removal, maximum-intensity z-projection, and automated binarization, a size filter was applied for objects and microglial shape descriptors were obtained (ImageJ – solidity, perimeter).

#### Immunostaining of free-floating brain slices

Five to six free-floating sections taken every 12th brain sections of 8-9 WT, *P2ry6*^*−/−*^, P301S^+/+^ and *P2ry6*^*−/−*^: P301S^+/+^ mice were used for immunohistochemistry. Sections were rinsed three times in PBS and incubated with 20% methanol, 3% hydrogen peroxide in PBS for 30 min at room temperature for quenching endogenous peroxidase activity. Sections were rinsed 3 times in 0.3% Triton-X in PBS (PBS-T) and were subsequently incubated overnight with shaking at 4°C with either NeuN or AT8 primary antibodies (NeuN; 1:500, Millipore, mab377; AT8; 1:700, Thermo Scientific, MN1020). Primary antibody incubation was extended for 2 more h at RT with shaking. Following 3 washes in PBS-T, sections were then incubated for 1 h at RT with the goat anti-mouse biotinylated secondary antibody (Vector Laboratories, BA-9200) diluted 1:250 in PBS-T. Subsequently, sections were washed 3 times in PBS-T and incubated with ABC Elite kit mix (Vectastain ABC Kit (Standard), PK-6100) for 30 min, following manufacturer’s instructions, and washed 3 times with PBS-T. The immunostaining was visualized with diaminobenzidine (DAB Vectastain kit (Vector). (DAB Peroxidase Substrate Cat. No. SK-4100; Vector Laboratories)) until desired stain intensity developed, approximately 2 min at RT. The sections were then quickly washed with 0.1% sodium azide - PBST and rinsed 3 times with PBST. Sections were then mounted on glass slides and dried in 37°C oven. Following dehydration of tissue sections in ascending concentration of alcohols, they were cleared in xylene and coverslipped with DPX (a mixture of distyrene, a plasticizer, and xylene). The tissue sections were finally scanned using a Zeiss Axioscan Z1 slide scanning microscope.

#### Image analysis of free-floating brain slices’ immunostaining

For image analysis and quantification of NeuN density, the Halo image analysis platform was used. First, the whole brain coronal slice was first highlighted and the perirhinal cortex (PRh) or motor cortex contour was outlined. NeuN-positive neurons in the corresponding outlined area were identified using the Indica labs algorithm, with few modifications (Image zoom: 0.4; Minimum tissue OD: 0.007; Nuclear contrast threshold: 0.53; Minimum nuclear OD: 0.695; Nuclear size: 26,717.717; Minimum nuclear roundness: 0.298; Nuclear segmentation aggressiveness: 1). Data are presented as neuronal density per mm^2^.

### Quantification and statistical analysis

#### Statistical analysis

In the *in vivo* studies, each data point represents either one field of view (Figures 1A–1D), one Iba1+ cell (Figures 1F–1G), or one animal (all other figures). In the *in vitro* studies, bars represent mean and SEM of N = 3-5 independent experiments performed in triplicate. Statistical significance for experiments with more than two groups was analyzed by two-way ANOVA with Tukey-corrected post hoc comparisons, except in Figures S5D–S5I where we compared periphery versus center for each group and therefore used one-way ANOVA with Bonferroni post hoc comparisons. Statistical differences in Figure 4G and 4H comparing two groups were calculated by using a two-tailed Student’s t test. Statistical differences in Figure S3 comparing two groups (LPS-treated WT versus LPS-treated P2Y6−/− microglia) were calculated by multiple unpaired t test followed by Holm-Sidak multiple comparisons test. All experiments were analyzed using GraphPad Prism 6 (GraphPad software). Graphical data were shown as individual data points, including mean values with error bars indicating SEM P values of ^∗^ p < 0.05, ^∗∗^p < 0.01, ^∗∗∗^p < 0.001, ^∗∗∗∗^p < 0.0001 indicated significant differences between groups. For each graph, all genotypes were compared, and if there is no marker of significance on the graph, then any difference was not significant. For each experiment and graph, statistical details including the statistical test used, the exact value of n, what n represents (field of view, number of cells, number of animals per genotype, etc.) as well as dispersion and precision measures (mean, SEM, etc.), can be found in each figure legend.

## Data Availability

Microscopy, behavioral, and flow cytometry data reported in this paper will be shared by the lead contact upon request. This paper does not report original code. Any additional information required to reanalyze the data reported in this paper is available from the lead contact upon request.

## Supplemental information

Document S1. Figures S1–S8

Document S2. Article plus supplemental information
