# Supplementary material for: The microglial P2Y6 receptor mediates neuronal loss and memory deficits in neurodegeneration
Source: Cell Rep. 2021 Dec 28;37(13):110148. doi: 10.1016/j.celrep.2021.110148 (PMC8733854; doi:10.1016/j.celrep.2021.110148)
Supplement: Document S1. Figures S1–S8 [file mmc1.pdf]

Cell Reports, Volume *37*

Supplemental information

The microglial $P2Y_6$ receptor mediates neuronal loss and memory deficits in neurodegeneration

Mar Puigdellívol, Stefan Milde, Anna Vilalta, Tom O.J. Cockram, David H. Allendorf, Jeffrey Y. Lee, Jacob M. Dundee, Katryna Pampuščenko, Vilmante Borutaite, Hugh N. Nuthall, Jack H. Brelstaff, Maria Grazia Spillantini, and Guy C. Brown

Supplementary Figure 1

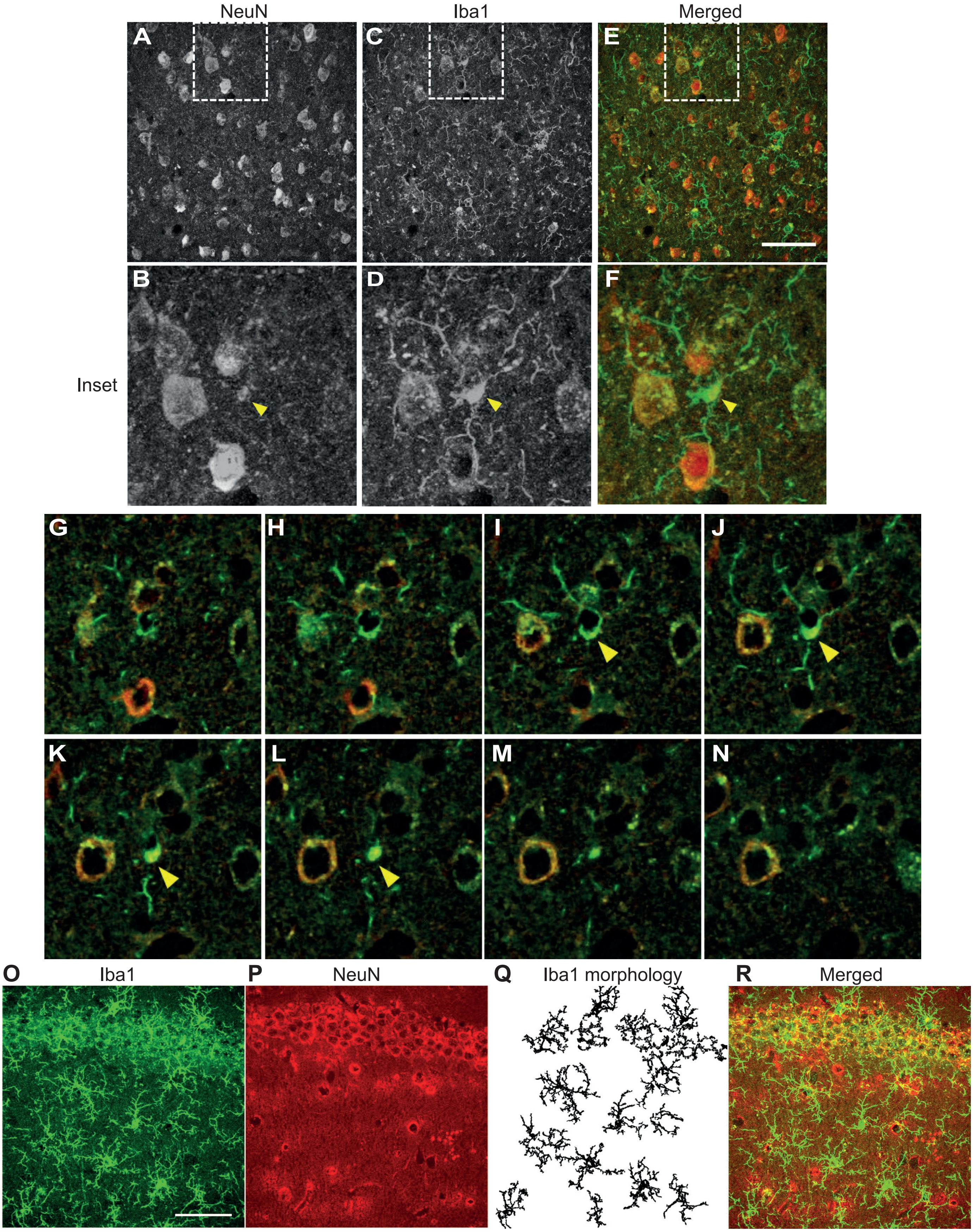

Figure S1. NeuN internalization within microglia in the prefrontal cortex and the hippocampus of Aβ-injected wild-type mice. Representative image of Iba1-positive microglia within NeuN-positive material ingested in coronal sections of prefrontal cortex following i.c.v injection of Aβ in wild-type (WT) mice. **A**, **C**, **E**, Larger field of prefrontal cortex region imaged for NeuN (**A**, gray scale), Iba1 (**C**, gray scale) and images merged (**E**, NeuN (neurons, red) and Iba1 (microglia, green)). Scale bar: 50 µm. **B**, **D** and **F**, Insets of A, C and E, respectively, providing a larger image of a single Iba1-positive (green) microglial cell with NeuN-positive material (red) inside (overlap yellow). **G-N**, Consecutive Z stacks of (F) showing that NeuN puncta colocalising Iba1 appeared in contiguous z-slices (yellow arrows, I-L). Yellow arrows indicate NeuN internalisation within microglia. **O**, **P**, **R**, Larger field of hippocampal region CA1 imaged for Iba1 (**O**, microglia, green), for NeuN (**P**, neurons, red), and these images merged (**R**). Scale bar: 50µm. **Q**, Raw confocal image (O) was segmented into microglial cells colocalizing with NeuN and background, showing a range of microglia and their morphologies. Related to Figure 1.

Supplementary Figure 2

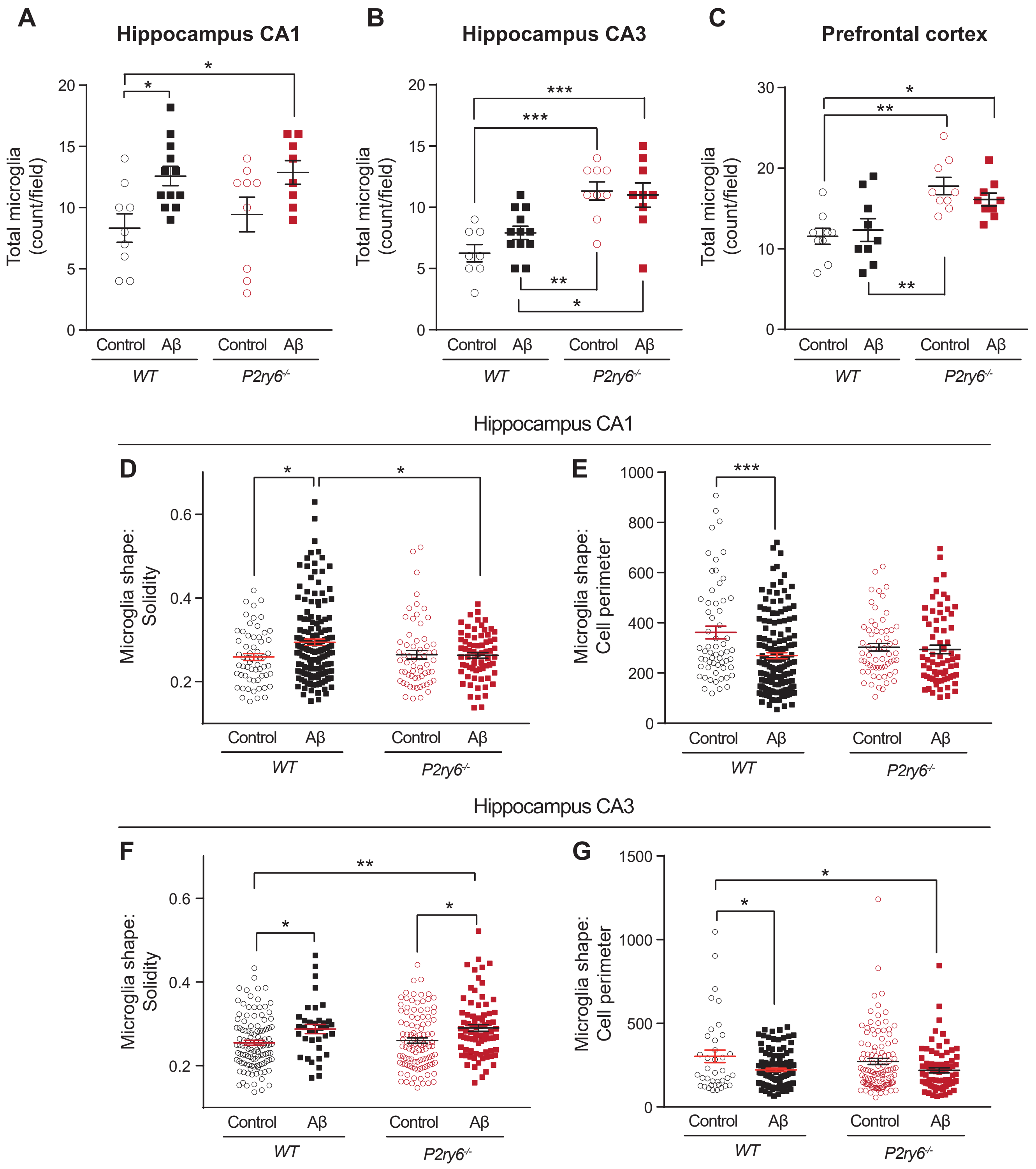

Figure S2. Aβ injection causes a mild morphological activation of microglia, which is little affected by P2ry6 knockout in mice. A-C, Analysis of microglial density in anatomically matched sections of hippocampal and cortical sections stained with anti-Iba1 antibody following i.c.v. injection of Aβ1-40 (Aβ) or PBS (control) in 10-month-old WT and P2ry6-/- mice. Total number of microglia per field of view in CA1 (**A**), CA3 (**B**) and prefrontal cortex (**C**). Each data point represents one field of view. Number of mice: WT-Aβ = 6, WT+Aβ = 6, KO-Aβ = 4, KO+Aβ = 4. Error bars indicate mean ± SEM. Statistical analysis was performed using two-way ANOVA with post hoc Tukey's multiple comparison test. *p<0.05, ** p<0.01, *** p<0.001. **D-G**, Analysis of microglial activation state by automated shape analysis in anatomically matched sections of hippocampus and prefrontal cortex following i.c.v. injection of Aβ1-40 (Aβ) or PBS (control) in 10-month-old WT and P2ry6-/- mice. Automated quantification of microglia shape solidity and perimeter in CA1 (**D** and **E**, respectively), and CA3 (**F** and **G**, respectively). Each data point represents one Iba1 + cell. Number of mice: WT-Aβ = 6, WT+Aβ = 6, KO-Aβ = 4, KO+Aβ = 4. Error bars indicate mean ± SEM. Statistical analysis was performed using two-way ANOVA with post hoc Tukey's multiple comparison test. *p<0.05, **p<0.01, ***p<0.001. For each graph, all genotypes ± Aβ were compared, and if there is no marker of significance on the graph, then any difference was not significant. Related to Figure 1.

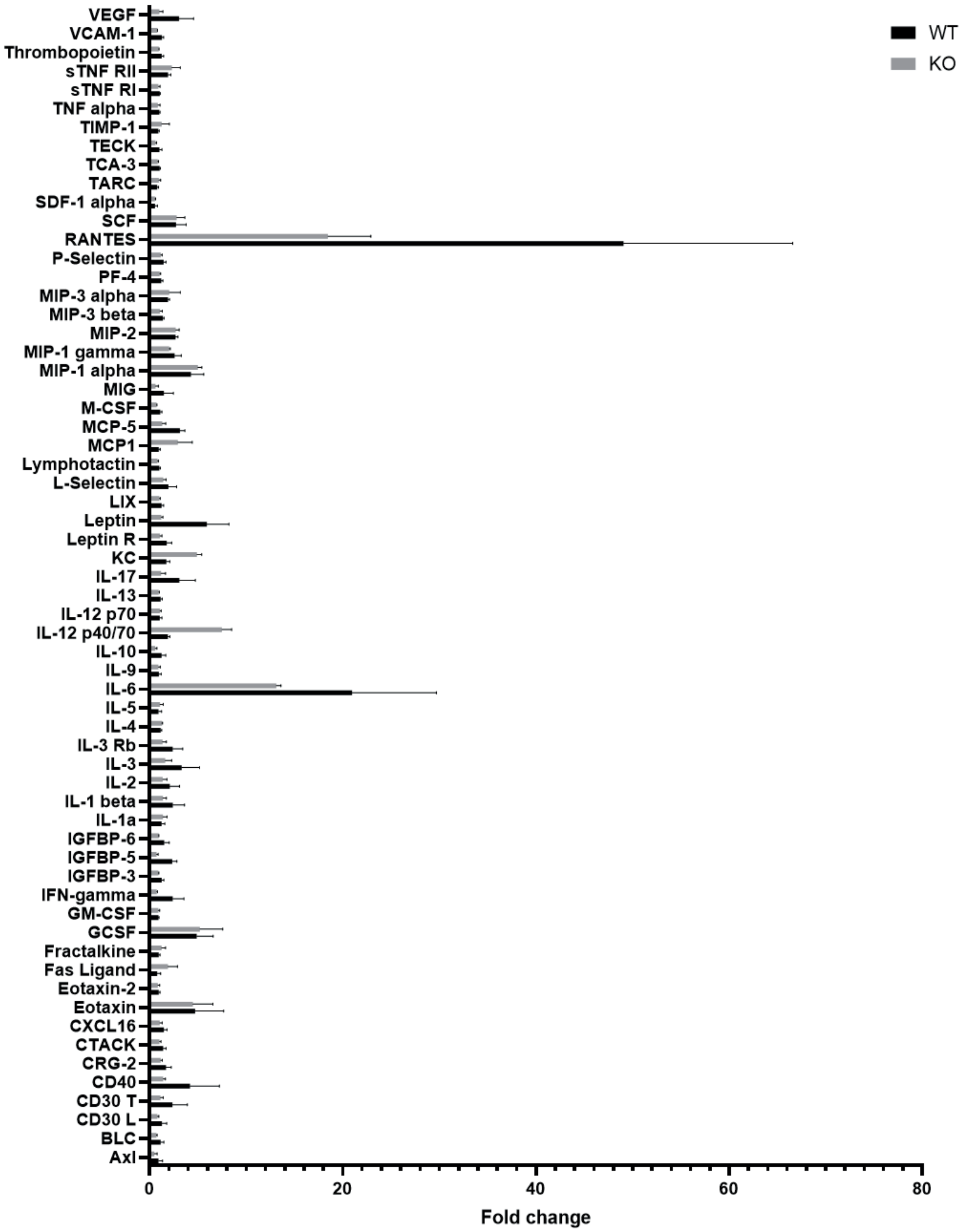

Figure S3. Similar microglia inflammatory profile of wild-type and P2ry6 knockout mice. ELISA array of 62 inflammatory cytokines and chemokines in the extracellular cell supernatant of primary microglia isolated from mixed glial cultures from wild-type and P2ry6-/- mice treated with ± 100 ng/ml lipopolysaccharide for 16 hours. Three independent experiments/genotype. The mean ± SEM of the LPS-induced fold change in each cytokine or chemokine is plotted, and the significance of these changes between wild-type and P2ry6-/- microglia were compared using multiple unpaired t-tests followed by Holm-Šidak multiple comparisons test. None of these differences in LPS-induced fold change between wild-type and P2ry6-/- microglia were statistically significant. Related to Figure 1.

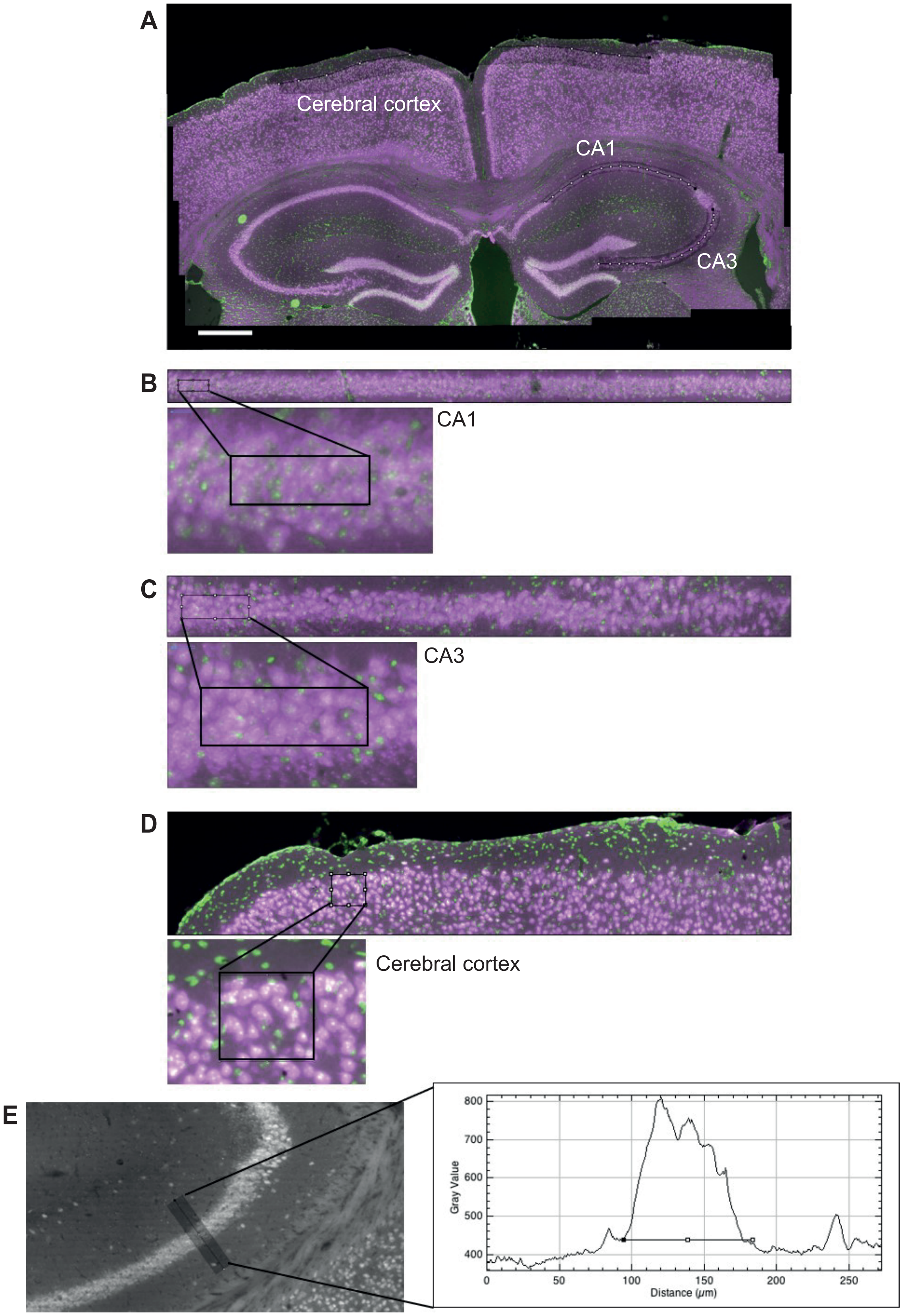

Figure S4. Details of neuronal density analysis in wild-type (WT) and P2ry6-/- mice following i.c.v injection of vehicle or Aβ. A, Larger view of mouse brain slice including cortical and hippocampal regions stained for nuclei (with DAPI, green) and for neuronal nuclei (with anti-NeuN antibody, magenta). Regions of interest for quantification in CA1, CA3 and cerebral cortex are shown. Scale bar, 500 μm. **B**-**D**, Representative images of CA1 hippocampus (**B**), CA3 hippocampus (**C**) or cerebral cortex (**D**). **E**, Representative image showing the stratum pyramidale histology of the CA1 hippocampus and histogram showing how width was estimated. Related to Figure 2.

Supplementary Figure 5

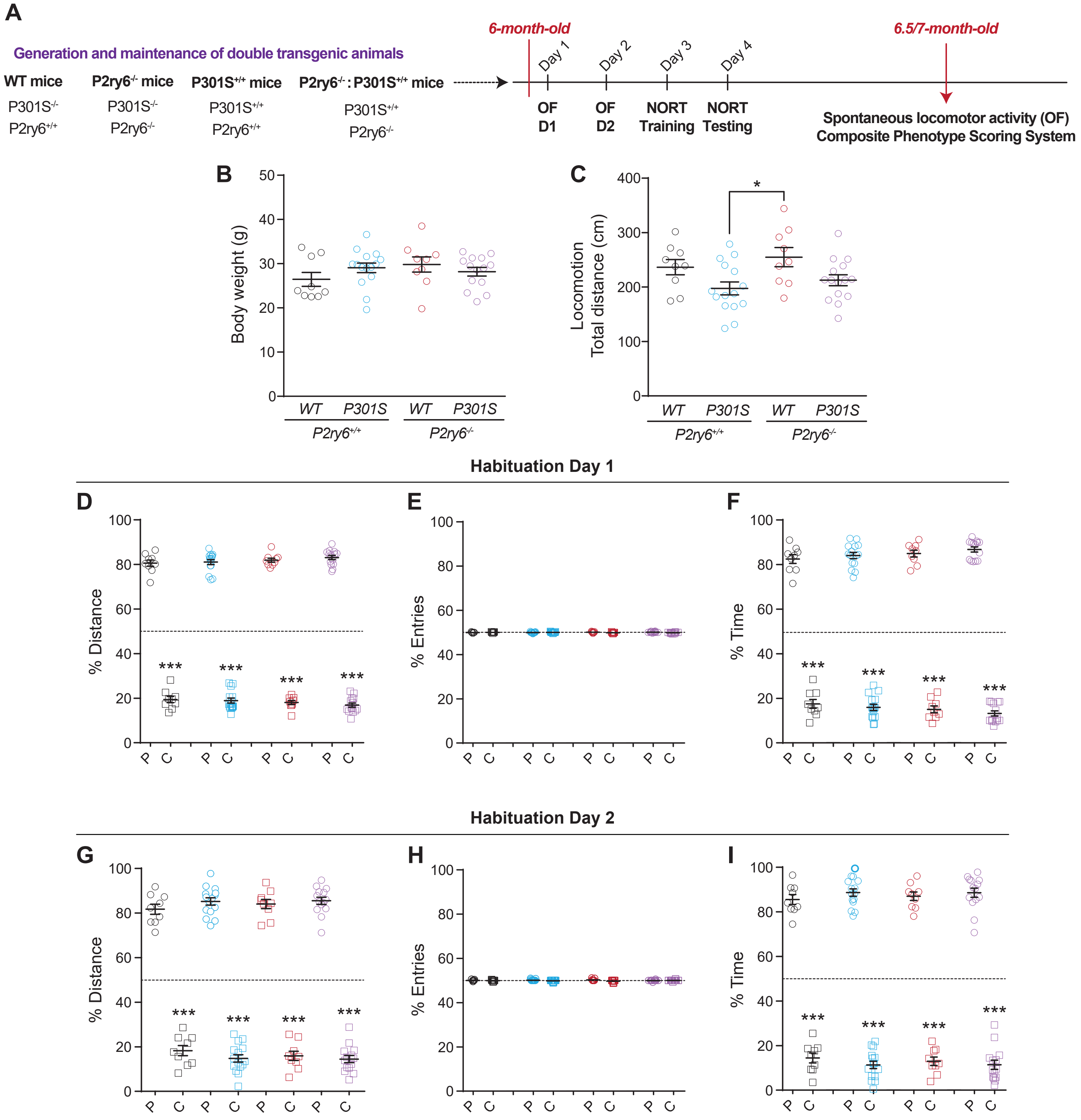

Figure S5. Body weight, locomotion, and anxiety and motivation in the open field are not significantly affected by P301S tau or P2ry6 knockout at 6 months of age. A, Timeline for generation and behavioral assessment of P2ry6+/+: P301S-/-, P2ry6+/+: P301S+/+, P2ry6-/-: P301S-/- and P2ry6-/-: P301S+/+ mice. At 6 months of age, mice were tested with open field (OF) and novel object recognition test (NORT, testing 24 hours after training). At 6.5-7 months of age, spontaneous locomotor activity and disease severity (hind limb clasping, ledge test, gait and kyphosis) was evaluated, followed by sacrifice of the mice and brain perfusion. **B**, Body weight of mice at 6 months of age. **C**, Spontaneous locomotion in open field at 6 months of age. Each data point represents one animal and error bars represent mean ± SEM. Data were analysed by two-way ANOVA with Tukey-corrected post-hoc comparisons. * $p<0.05$. For each graph, all genotypes were compared, and if there is no marker of significance on the graph, then any difference was not significant. **D-I**, Motivation and anxiety differences between genotypes were analyzed by measuring the percentage of distance (**D** and **G**), the percentage of entries (**E** and **H**) and the percentage of time spent (**F** and **I**) between the periphery (P) and the center (C) in the open field arena at the first (**D-F**) or second (**G-I**) day of habituation in the open field arena (15 minutes/day). Dashed lines indicate 50% chance level. Each data point represents one animal and error bars represent mean ± SEM. Statistical analysis was performed using one-way ANOVA with Bonferroni post hoc comparisons; ***$p<0.001$ compared with the periphery. Related to Figure 3.

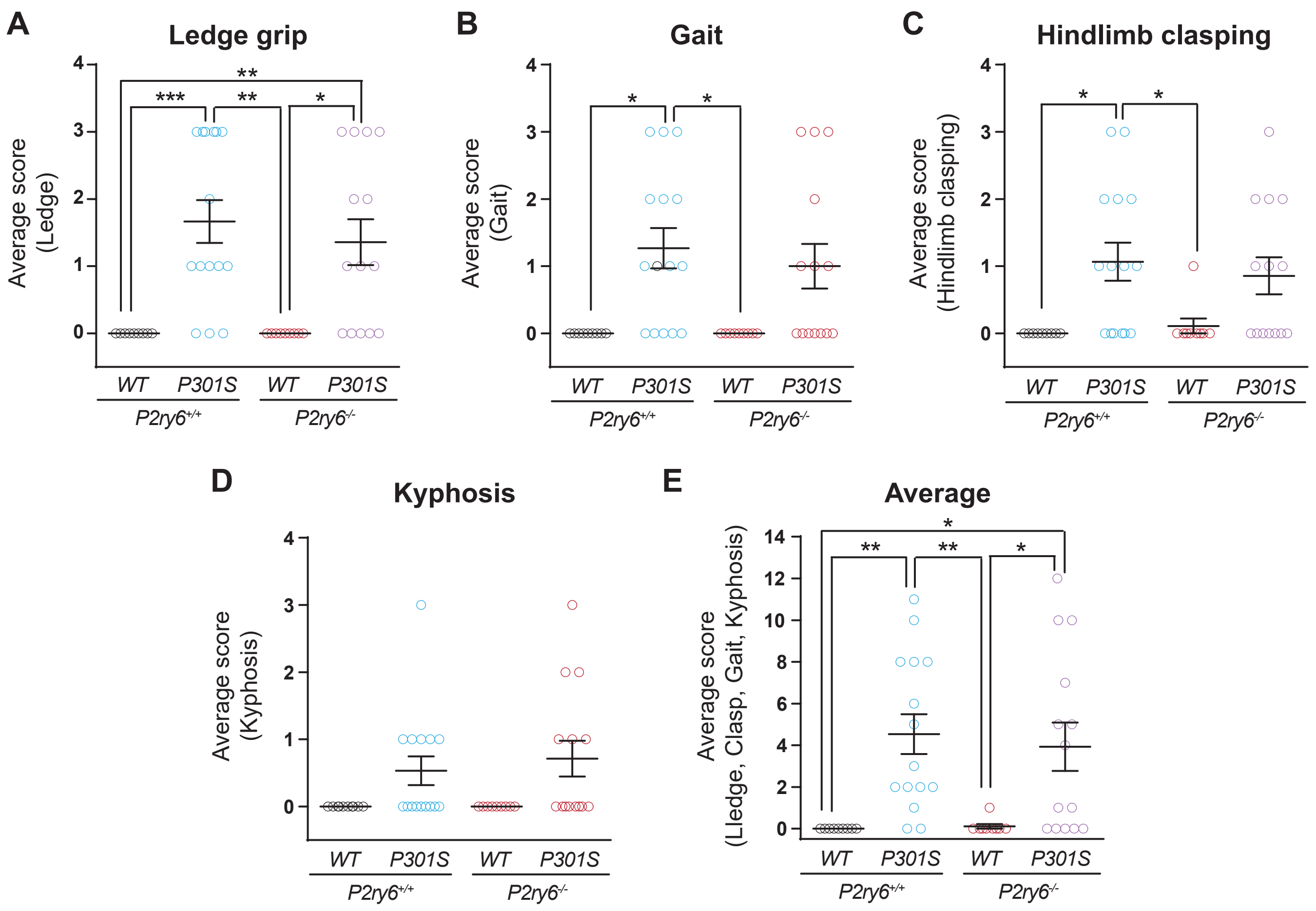

Figure S6. P301S tau mice develop motor deficits at 7 months, but these are not reduced in double transgenic (P2ry6-/-: P301S+/+ mice). Histograms showing motor function of wild-type (WT), transgenic P301S (P301S+/+), P2ry6 knockout (P2ry6-/-), and new double transgenic (P2ry6-/-: P301S+/+) mice at 7 months of age. **A**, Ledge. **B**, Gait. **C**, Hind limb clasping. **D**, Kyphosis. Data were analysed by two-way ANOVA with Tukey corrected post-hoc comparisons. *p<0.05, **p<0.01 and ***p<0.01. For each graph, all genotypes were compared, and if there is no marker of significance on the graph, then any difference was not significant. Related to Figure 3.

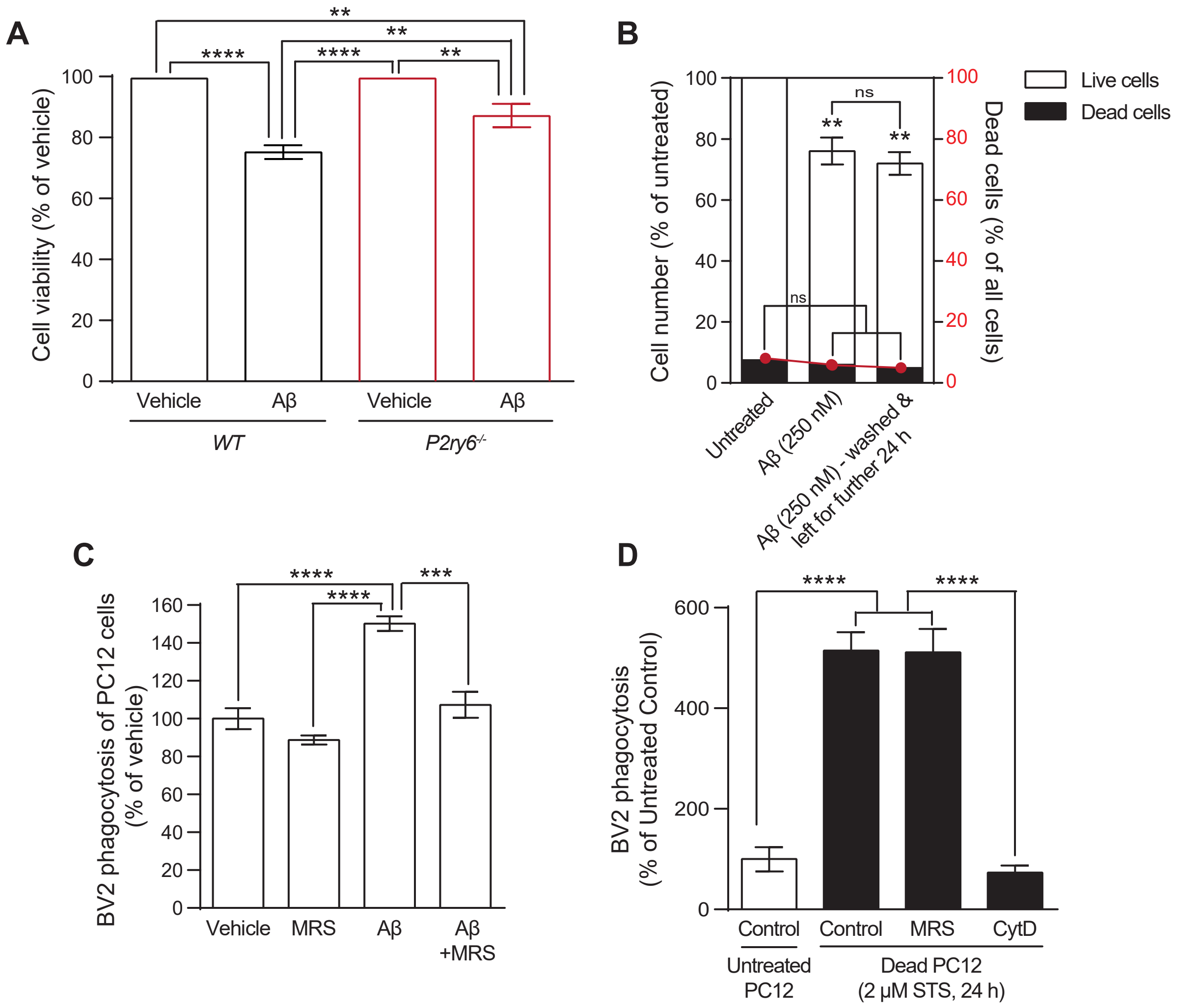

Figure S7. In vitro studies. A, Mixed neuronal-glial cultures from cerebella of wild-type (WT) or P2ry6-/- mice were treated for 3 days with ± 250 nM Aβ, then cell viability was measured by MTT assay. Data represent mean ± SEM (N=5-9). Statistical analysis was performed using two-way ANOVA with post hoc Tukey's multiple comparison test. ** p<0.01, **** p<0.0001. **B**, PC12 cells were treated ± 250 nM Aβ for 24 hours, and live and dead cells counted, to estimate proliferation and death (N=3). Another set of cells was washed and cultured for a further 24 hours, to check there was no delayed death. Aβ reduced PC12 cell proliferation, but did not induce any PC12 cell death. **p<0.01 compared with untreated. **C**, PC12 cells treated ± 250 nM Aβ for 24 hours were added to LPS-pre-treated BV-2 microglia ± 1 μM MRS2578 for 3 hours, then BV-2 phagocytosis of the PC12 assayed by flow cytometry (N=4). MRS2578 prevented the phagocytosis of Aβ-stressed PC12 cells, but not unstressed cells. *** p<0.001, **** p<0.0001. **D**, PC12 cells treated ± 2 μM staurosporine (STS, to induce cell death) for 24 hours were added to LPS-pre-treated BV-2 microglia ± 1 μM MRS2578 (MRS, to inhibit $P2Y_6R$) ± 1 μM cytochalasin D (CytD, to inhibit all phagocytosis) for 3 hours, then BV-2 phagocytosis of the PC12 cells was assayed by flow cytometry (N=3). The $P2Y_6R$ inhibitor had no effect on the phagocytosis of dead PC12 cells. Data represent mean ± SEM. Statistical analysis was performed using one-way ANOVA with post hoc Tukey's multiple comparison test. **** p<0.0001. Related to Figure 4.

Homozygous P301S (*P301S*+/+) mice (*P301S*+/+; *P2ry6*+/+) **X** (*P301S*-/-; *P2ry6*-/-) Homozygous P2ry6 knock-out (*P2ry6*-/-) mice

↓

Double heterozygous *P301S*+/-; *P2ry6*+/- mice (*P301S*+/-; *P2ry6*+/-) **X** (*P301S*+/-; *P2ry6*+/-)

↓

Final experimental animals:

(P301S+/+; P2ry6+/+)	(*P301S*+/+; *P2ry6*+/-)	***(P301S+/+; P2ry6-/-)***	(*P301S*+/+; *P2ry6*-/+)
(*P301S*-/+; *P2ry6*+/+)	(*P301S*-/+; *P2ry6*+/-)	(*P301S*-/+; *P2ry6*-/-)	(*P301S*-/+; *P2ry6*-/+)
(*P301S*+/-; *P2ry6*+/+)	(*P301S*+/-; *P2ry6*+/-)	(*P301S*+/-; *P2ry6*-/-)	(*P301S*+/-; *P2ry6*-/+)
(P301S-/-; P2ry6+/+)	(*P301S*-/-; *P2ry6*+/-)	***(P301S-/-; P2ry6-/-)***	(*P301S*-/-; *P2ry6*-/+)

Wild-type: *P301S-/-; P2ry6+/+*
P301S: *P301S+/+; P2ry6+/+*
P2ry6 knock-out: *P301S-/-; P2ry6-/-*
Double mutant: *P301S+/+; P2ry6-/-*

Figure S8. Schematic representation showing the breeding strategy for the generation of a new double transgenic mice expressing human mutant P301S tau and lacking the expression of P2Y6 receptor (P301S+/+; P2ry6-/-). Related to Figure 3.
